# Multi‐Mechanical Regulation of 3D Printed Triply Periodic Hyperbolic Surfaces via Fourier Synthesis‐Based Free Modeling

**DOI:** 10.1002/advs.202503694

**Published:** 2025-05-19

**Authors:** Yanhong Zhang, Junming Zhang, Zhimei Zhou, Yan Li, Shunai Che, Weidong Yang, Lu Han

**Affiliations:** ^1^ School of Chemical Science and Engineering Tongji University Shanghai 200092 China; ^2^ School of Aerospace Engineering and Applied Mechanics Tongji University Shanghai 200092 China; ^3^ School of Chemistry and Chemical Engineering State Key Laboratory of Composite Materials Shanghai Key Laboratory for Molecular Engineering of Chiral Drugs Shanghai Jiao Tong University Shanghai 200240 China

**Keywords:** 3D printing, Fourier synthesis, mechanical property, mortise‐tenon joints, triply periodic hyperbolic surface

## Abstract

Triply periodic hyperbolic surfaces (TPHSs) have attracted significant attention due to their exceptional lightweight and mechanical properties, which surpass those of other lattice structures. These advantages are primarily attributed to their unique periodic geometries and saddle‐shaped surface configurations. However, current structural design methods mainly rely on narrowband forward or multivariable inverse design strategies, which greatly limits the structural diversity and tunability of TPHSs, thereby hindering their further advancements in engineering applications. Herein, a hierarchical design method inspired by crystallographic Fourier synthesis is proposed, enabling the construction of arbitrary complex structures and the regulation of mechanical properties in multiple ways. By utilizing this approach, any structural types of TPHSs, including the most appealing primitive, gyroid, diamond‐like surfaces and their structural variants, are additively manufactured. This method enables precise manipulation of fine structural features to optimize 3D stress fields, significantly enhancing overall stiffness and strength. Moreover, this method facilitates the design of unbalanced TPHSs with rod‐like characteristics, enabling structural assembly through mortise‐tenon joints, which greatly expands the construction methodologies for such structures. This research substantially extends the design space of TPHS‐based structures and paves the way for their widespread application in advanced engineering contexts.

## Graphical abstract

Arbitrary structural regulations of triple periodic hyperbolic surfaces are achieved using mathematical Fourier construction and 3D printing technology, enriching the geometric forms and achieving optimized mechanical behaviors.

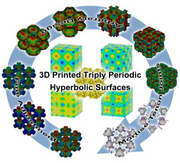

## 1 Introduction

Triply periodic hyperbolic surfaces (TPHSs) are intricate 3D geometries formed by infinite, non‐intersecting, and periodically arranged surfaces with negative Gaussian curvature. These structures have been widely observed in various biological systems, such as cell membranes, butterfly wing scales, beetle exoskeletons, and are also commonly found in amphiphilic molecular self‐assembly systems, including microphase‐separated block copolymers and lyotropic liquid crystal structures of surfactants.^[^ ^1^ ^]^ To date, TPHSs are considered to be among the most complex periodic structures discovered so far. Among these, the gyroid (G), diamond (D), and primitive (P) surfaces attracted great attention as the most notable and important examples.

In recent years, the exploration of the properties and applications of TPHSs continues to be an active area of research across multiple disciplines. Particularly, TPHSs have been used as models for designing lightweight mechanical materials due to their unique mechanical responses.^[^ ^2^ ^]^ TPHSs show excellent strength‐weight ratio, high energy absorption efficiency, and unique deformation mechanism, particularly, these structures avoid the stress concentration at the sharp edges or nodal sites as the typical cubic and lattice structures.^[^ ^3^ ^]^ These features make TPHSs stand out among other mechanical structures and become excellent candidates for the next generation of lightweight mechanical structures for engineering applications.^[^ ^2a^ ^]^ Benefit from the recent advances in additive manufacturing, the mechanical properties of TPHS structures have been extensively investigated, including yield strength,^[^ ^4^ ^]^ effective elastic modulus,^[^ ^2^, ^5^ ^]^ Poisson's ratios,^[^ ^6^ ^]^ energy absorption,^[^ ^2^, ^6^, ^7^ ^]^ etc.^[^ ^2^, ^7^, ^8^ ^]^ which have been widely envisaged in the field of biomedical, automotive, and aerospace engineering, etc.^[^ ^9^ ^]^

The geometrical design and structural parameters of materials play a crucial role in determining their mechanical behaviors and applications.^[^ ^10^ ^]^ For traditional metamaterials such as octagonal trusses, cubes, body‐centered cubes, truncated cubes, etc., three primary methods have been employed to tailor unit cell configurations: the hybrid method involves the interconnection of existing structural units to form new architectures;^[^ ^11^ ^]^ the curved geometry design optimizes structural performance within individual cells by introducing non‐linear forms such as U‐shaped, V‐shaped, or spherical units;^[^ ^12^ ^]^ and the enhanced design focuses on improving mechanical performance by incorporating strengthening features, including the adjustments to element thickness, modification of joint topology, and the strategic addition of reinforcing elements such as bars and ribs.^[^ ^13^ ^]^ For TPHS structures, recent studies have shown that different topological types,^[^ ^14^ ^]^ relative density,^[^ ^15^ ^]^ unit cell size,^[^ ^5^, ^16^ ^]^ and orientations^[^ ^17^ ^]^ lead to diverse mechanical performances. In addition, various structural design methods have been proposed to meet the needs of different applications, such as graded,^[^ ^2^, ^18^ ^]^ heterogeneous,^[^ ^16^, ^19^ ^]^ multiscale,^[^ ^20^ ^]^ design of external shapes,^[^ ^21^ ^]^ thin‐walled structures,^[^ ^22^ ^]^ composite structures,^[^ ^23^ ^]^ etc. These methods have been proven efficient in load resistance and energy absorption. However, current design methodologies typically rely on simplified level‐set equations, which limit TPHS applications to predetermined categories and restrict the ability to freely modulate surface characteristics. This results in a significantly constrained design space, enabling only rudimentary adjustments rather than comprehensive optimization of structural configurations, and the reverse design by machine learning is often poor in multi‐objective optimization. A general construction method for arbitrary design of TPHS structures with controllable curvatures and mechanical properties remains challenging, significantly limiting the applications of these hyperbolic surface structures.

Herein, we report the arbitrary design and multi‐mechanical regulation of 3D printed TPHSs constructed by Fourier synthesis‐based structure modeling, by which the diversified mechanical properties and deformation mechanisms, as well as the geometrical shape required for specific mechanical scenarios, can be extensively regulated. This method is based on the calculation of periodic structure, i.e., the spatial density distribution in 3D, by the linear superimposition of sine and cosine waves corresponding to different Bragg reflections associated with the crystal space group symmetries.^[^ ^24^ ^]^ This approach is capable of incorporating all space group symmetries and allows for the construction of arbitrary periodic structures with smooth surface representations by simply adjusting the amplitudes and phases of sine or cosine waves. All models were then 3D printed and their mechanical properties were measured by compression tests. The construction of the three most attractive TPHSs, G, D, and P surface structures and their arbitrary adjustment and mechanical optimization of surface positions were achieved. We also demonstrated the possibility of modeling new types of arbitrary hyperbolic structures. Due to the controllability of the structure, we further designed a mortise‐tenon structure to realize the disassembly and assembly of the hyperbolic surfaces, which also implies the possibility of applying these structures on an architectural scale. Finite element (FE) simulations were applied to reveal the stress distribution during the compression process.

## 2 Results and Discussion

### 2.1 Fourier Construction of TPHS Models

**Figure 1:**
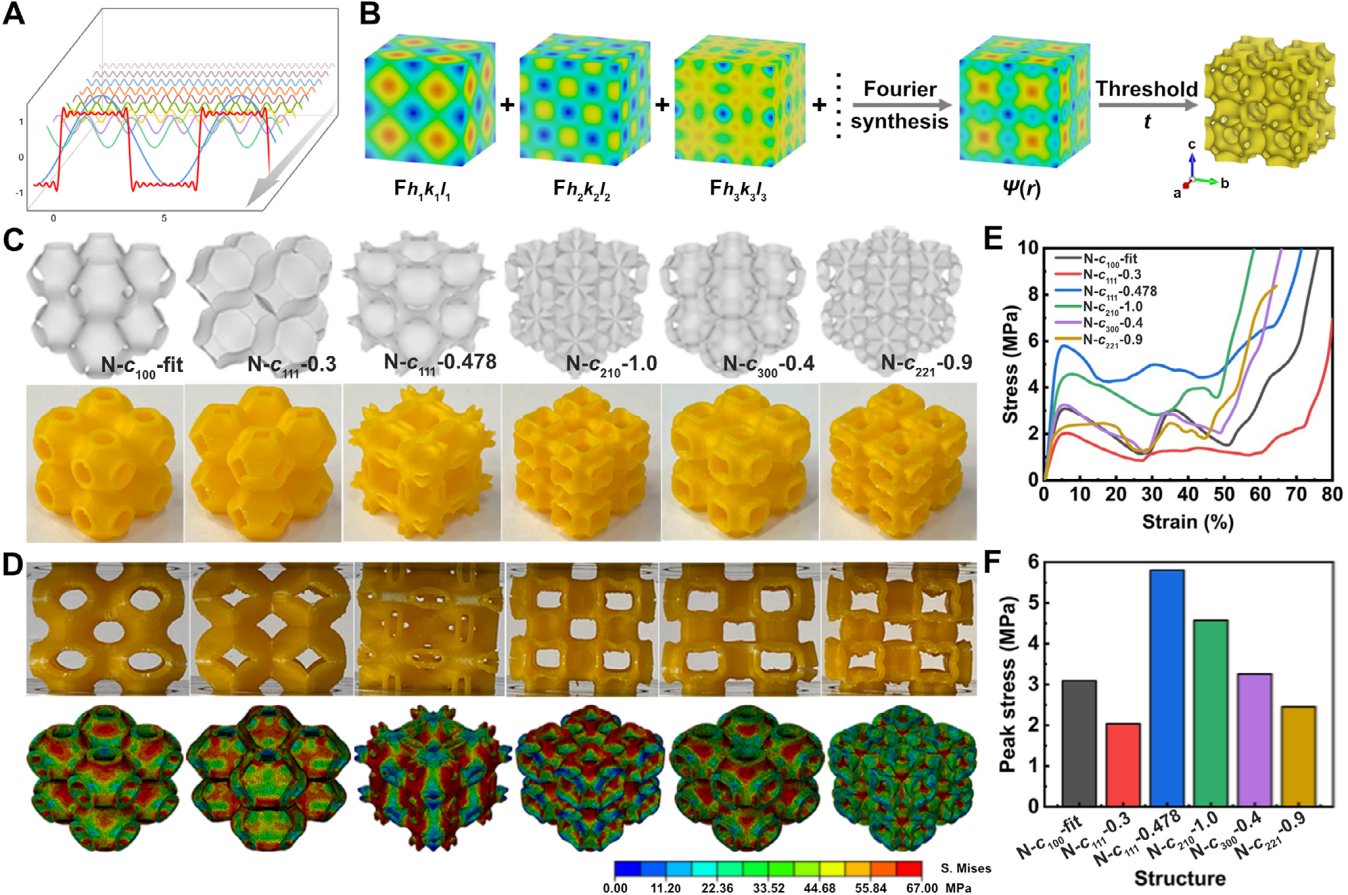
The structural design of TPHSs based on Fourier synthesis. A) Schematic drawing of the principle of Fourier synthesis. B) Construction of TPHS structures. C) Various structures were constructed based on *Pm* $\bar{3}$ *m* symmetry by adjusting corresponding Fourier components. D) Snapshots and FE simulations of corresponding structures under compressive load. E) Stress‐strain curves and F) Peak stress of each structure.

Extensively employed in mathematical and engineering fields, the Fourier transformation decomposes a periodic function into various sine and cosine components, and the reconstruction of the original periodic function from these components is known as Fourier synthesis (**Figure** **1**A). Each sine or cosine wave corresponds to a periodic density distribution in the periodic structure, namely the ***k*** Miller index Bragg reflection. By applying different space group symmetries with distinctive periodicities, the periodic density distribution Ψ(*r*) can be modeled by the Fourier series using the structure factor *F*(***k***) with a given reciprocal lattice vector ***k*** and the phase shift *α*(***k***)^[^ ^24^ ^]^

(1) $$\Psi \left(r\right)=\sum_{k}F\left(k\right)\cos\left[2\pi k\cdot r-\alpha \left(k\right)\right]=t$$

The isosurface can be obtained by setting a threshold value *t* in Ψ(*r*) (Figure 1B). Truncating the Fourier series to the leading term yields the simple level surface equations. However, such equations exhibit limited capability in adjusting structural features, and material property adjustments are significantly constrained. By integrating higher‐order Fourier components into structural modeling, design diversity and adjustability can be substantially improved. This allows for the generation of different hyperbolic structures by leveraging appropriate space group symmetries and their corresponding reflections within the lattice framework of a given space group. Furthermore, this method can be applied to all 230 space group symmetries, enabling the creation of arbitrary periodic smooth geometries.

### 2.2 Structural Construction and Regulation

#### 2.2.1 Design of TPHSs

Inspired by this idea, structures with tailored mechanical properties and deformation modes can be easily designed. The structural models are established by performing a Fourier synthesis following the calculation equations from the literature (see Equation S1, Supporting Information for details).^[^ ^25^ ^]^ Taking the space group symmetry of simple cubic *Pm* $\bar{3}$ *m* as an example, five *hkl* Bragg reflections, namely 100, 111, 210, 300, and 221 were introduced. Each reflection has a significant effect on the final structure according to its periodicity, amplitude, and phase. The larger the amplitude, the greater the influence of the reflection on the final structure. By setting the coefficient (amplitude) for the 100 reflection with *c*_100_ = 1, and regulating the coefficient of other reflections, the contributions of corresponding *hkl* reflection to the final density distribution in the structure can be fine regulated and a variety of TPHSs with distinct geometrical features can be obtained. For clearer observation of experimental deformation behaviors and stress distributions in FE simulations, we adopted a two‐layer structure with larger unit cell size. The porosity of the structures was determined by calculating the ratio of the mass of the target hyperbolic structure to the mass of a solid cube with the same dimensions. It is worth noting that, the combination of Fourier components may cause discontinuity in 3D density distribution, i.e., the formation of discrete structure, which can be solved by adjusting the proportions of each Fourier term. The evolution of the structures is clearly shown in Movies S1–S4 (Supporting Information).

According to this scheme, various structures with different combinations of Fourier components during the evolutions were realized by Fused Deposition Modeling (FDM) 3D printing and tested by quasi‐static compression (Figure 1C,D). The threshold value *t* was first set to 0 to reveal the influence of the Fourier coefficients on each structure as shown in Table S1 (Supporting Information). The samples were named as N‐*c_hkl_*‐coefficient, using coefficients corresponding to the *hkl* reflection with the maximum amplitude. As shown in Figure 1C, the N‐*c*_100_‐fit showed the same structural feature as the conventional P surface. While the regulation of Fourier terms and coefficients led to variation in structural characteristics. N‐*c*_111_‐0.3 exhibited similar structure with Kelvin foam with flatter local regions, while N‐*c*_221_‐0.9 showed sharper corners. N‐*c*_210_‐1 and N‐*c*_300_‐0.4 maintained the characteristics of negative curvature but with different pore shapes and N‐*c*_111_‐0.478 presented a completely different morphology. However, the crystal symmetry of these structures remained unchanged due to the restriction of the space group.

The stress‐train curves showed that all the structures experienced similar mechanical responses in four stages, namely, the initial linear elastic stage, the nonlinear elastic‐plastic stage, the stress fluctuation or plain stage after reaching the peak stress and the final densification (Figure 1E). It was found that the regions with negative Gaussian curvature facilitate the dispersion of stresses and the reduction of these regions weakens the structural strength, e.g. N‐*c*_111_‐0.3. When sharp corners emerged during structural changes, they led to diminished mechanical performance due to stress concentration at these corners, e.g. N‐*c*_221_‐0.9. Conversely, structures that retain features of negative Gaussian curvature and exhibit more material distribution along the load direction demonstrated columnar strengthening, e.g. N‐*c*_111_‐0.478 and N‐*c*_210_‐1.0. The stress distribution and structural mechanics of the structures were further justified by FE simulations, showing good agreement in stress distributions and the stress‐strain curves (Figure 1D; Figure S1, Supporting Information). To simplify the modeling process and improve computational efficiency, material damage and failure were not considered in most of the simulations; only plastic deformation was taken into account as the reduction in the material's load‐bearing capacity is primarily due to plastic yielding. In the N‐*c*_221_‐0.9 structure, severe stress concentration appeared at the corners caused a deterioration in mechanical properties. In contrast, the N‐*c*_111_‐0.478 showed enhanced mechanical properties, characterized by vertical stress bands and stretching‐dominated deformation along the load direction. The remaining structures underwent random wall tensile deformation. By analyzing the peak stress from stress‐strain curves, we plotted the results in Figure 1F. Compared to the conventional leading‐term level surface approximation for P surface structures (N‐*c*_100_‐fit), the N‐*c*_111_‐0.478 structure achieved an 86.21% improvement in peak stress through Fourier construction. It can be observed that the more uniform vertical stress distribution in compressive N‐*c*_111_‐0.478 structure contributed to higher peak stress and superior energy absorption capability. In addition, more structural models and mechanical characterizations are presented in Figure S2 (Supporting Information), demonstrating the advantages and design freedom of Fourier synthesis in terms of structural optimization and performance modulation.

#### 2.2.2 Regulation of G, D, P Surface Structures by Fourier Synthesis

**Figure 2:**
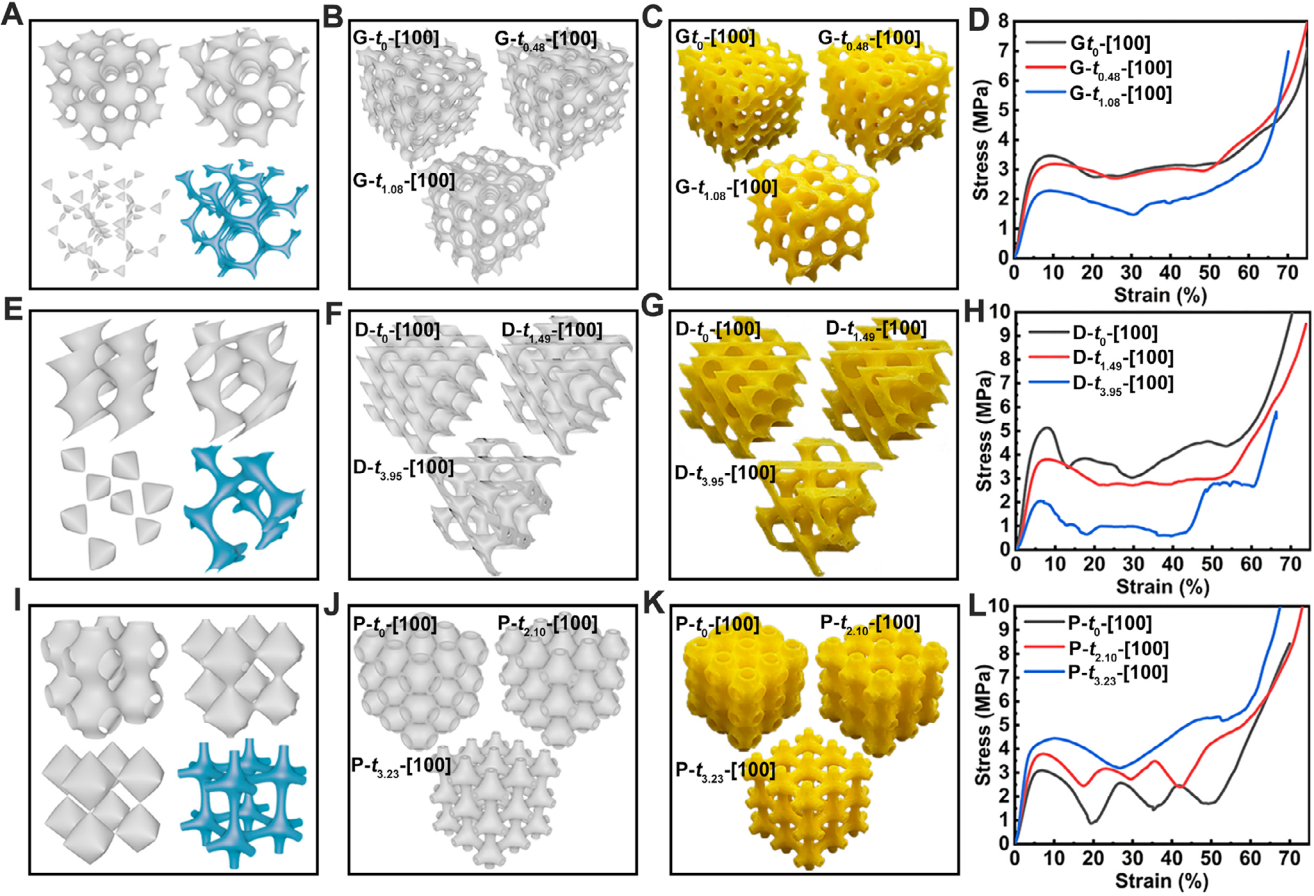
Structure optimization and the effect of threshold value on the structural mechanical properties in [100] direction. A) G leading‐term structures with different threshold values (gray) and high threshold value structures with second‐order Fourier term optimized (blue). B) Structural models and C) Physical models of G‐derived structures with second‐order Fourier term optimized under different threshold values. D) Stress‐strain curves of G‐related structures. E) D leading‐term structures with different threshold values (gray) and high threshold value structures with second‐ and third‐order Fourier term optimized (blue). F) Structural models and G) Physical models of D‐derived structures with second‐ and third‐order Fourier term optimized under different threshold values. H) Stress‐strain curves of D‐related structures. I) P leading‐term structures with different threshold values (gray) and high threshold value structures with second‐order Fourier term optimized (blue). J) Structural models and K) Physical models of P‐derived structures with second‐order Fourier term optimized under different threshold values. L) Stress‐strain curves of P‐related structures.

We applied this method to modulate the three most appealing TPHSs, namely G, D, and P surface structures. When constructing these structures using conventional level set surface equations, the pinch‐off problem often occurs at “large nodes” and “thin struts,” especially at threshold value *t* increases, leading to discontinuous structures and making it impossible to fabricate structures with low volume fraction (**Figure** **2**A,E,I). We determined the threshold values for level set G, D, and P structures to be *t*_G_ ∈ [−1.41, 1.41], *t*_D_ ∈ [−4.2, 4.2] and *t*_P_ ∈ [−1.9, 1.9]. However, the pinch‐off problem in TPHS structures can be easily optimized by regulating the strength of Fourier terms to balance weak and excessive parts (blue‐colored models shown in Figure 2A,E,I). The corresponding equations as shown in Equations S2 and S3 (Supporting Information). By introducing Fourier terms corresponding to the 110 and 222 reflections for space group *I*4_1_32; 111, 331, and 511 reflections for space group *Fd* $\bar{3}$ *m*; 100 and 111 reflections for space group *Pm* $\bar{3}$ *m*, respectively, we achieved more homogeneous structures with improved threshold ranges. The threshold values of D and P surface structures increased to *t*_D’_ ∈ [−5.8, 5.8] and *t*_P’_ ∈ [−3.9, 3.9] due to the increased homogeneity, while *t*_G’_ remains similar since the G surface already possesses the most homogeneous structural characteristics. To investigate the influence of the threshold value *t* on structural mechanical properties, G‐, D‐ and P‐derived structures under different threshold values were modeled and fabricated by 3D printing (Figure 2B,C,F,G,J,K) and the parameters of all models are listed in Table S2 (Supporting Information). Notably, since the boundary effects on mechanical properties diminish as the number of unit cell layers increases. Therefore, in the detailed mechanical property comparisons described in this section, the three‐layer unit cell models were employed. Considering that the structures were modeled by taking the volume enclosed by the surface corresponding to two different threshold values, with *t* denoting the average value of these two thresholds. Each model was named based on the corresponding average threshold *t* and loading direction. As the threshold increased, the structure became thinner, adopting strut‐like characteristics. Subsequent compression tests were conducted to obtain their mechanical curves (Figure 2D,H,L). Based on the stress‐strain curves, the mechanical properties of G‐ and D‐derived structures were weakened as the structure became thinner, while the P‐derived structures became stronger. This is due to the change in deformation modes: for G‐ and D‐derived structures, deformation shifted from wall stretching to strut bending; for P‐derived structures, it changed from wall stretching to strut stretching. These observations were further verified by later deformation process and FE simulations. Notably, the D‐*t*_0_‐[100] structure exhibited the highest peak strength but was also most sensitive to variations in the threshold values.

**Figure 3:**
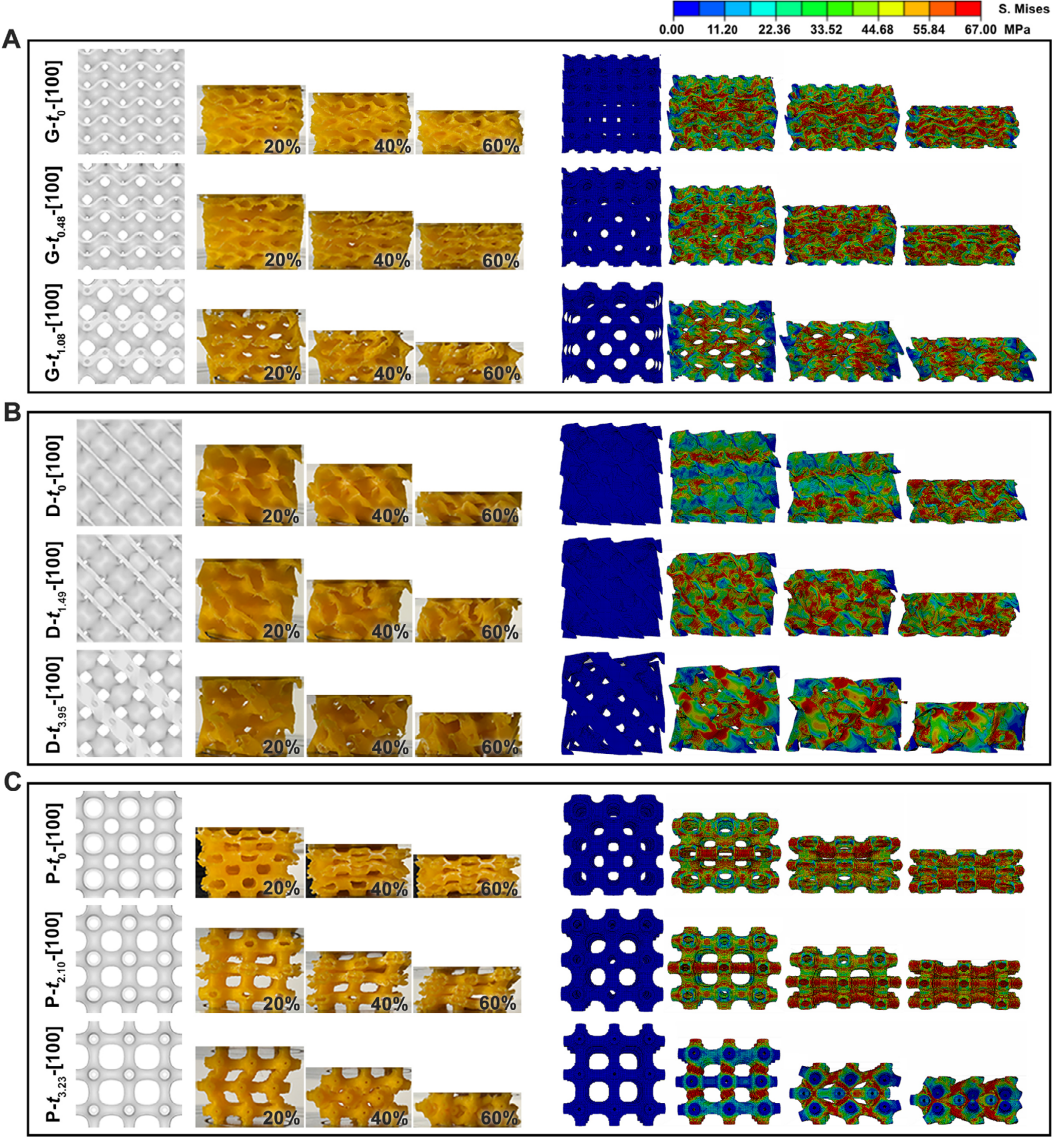
3D printed G‐related structures under compressive load and corresponding cases from FE simulations. A) G‐related structures. B) D‐related structures and C) P‐related structures. The colors indicate the local level of stress (von Mises stress).

By observing the snapshots during the compression process, the structural deformation mechanism was changed with the threshold values. It can be seen from **Figure** **3**A that G‐*t*_0_‐[100] and G‐*t*_0.48_‐[100] deformed with the stretching of walls, while G‐*t*_1.08_‐[100] showed the struts bent around the nodes to accommodate the applied loads, the deformation mechanisms changed from stretching‐dominated to bending‐dominated when the threshold value increased to 1.08. The corresponding FE simulations showed that the stress was widely and evenly distributed along the wall in G‐*t*_0_‐[100] and G‐*t*_0.48_‐[100], while in G‐*t*_1.08_‐[100], the stress was mainly concentrated on the inclined struts accompanied with shearing force. The deformation behaviors and stress distribution of D‐derived structures were also similar (Figure 3B). When the threshold value increased to 3.95, the structure (D‐*t*_3.95_‐[100]) underwent bending‐dominated deformation. However, the deformation behaviors of P‐derived structures were different (Figure 3C). As the threshold value increased, the deformation mechanism of the structure changed from the collapse of nodes (P‐*t*_0_‐[100] and P‐*t*_2.10_‐[100]) to the buckling of struts (P‐*t*_3.23_‐[100]) since the struts are all horizontal or vertical. This phenomenon is consistent with the FE simulated stress distribution that there were horizontal stress bands in P‐*t*_0_‐[100] and P‐*t*_2.10_‐[100], while vertical stress bands in P‐*t*_3.23_‐[100]. The comparison of stress‐strain curves between experimental data and simulation results of these samples are illustrated in Figure S3 (Supporting Information).

In addition, the effect of the threshold value on the structural mechanical properties in the other two characteristic directions, [110] and [111], were also investigated. For the structures in [110] direction (Figure S4, Supporting Information), there is an optimal threshold value of 0.48 and 1.49 for G‐ and D‐derived structures to obtain better peak stress, respectively, while P‐derived structures are weakened with the increased threshold values. These differences are attributed to the angle between the struts of the thinner structures and the loading direction. In G‐*t*_0.48_‐[110] and D‐*t*_1.49_‐[110], the buckling of vertical cylindrical surfaces was observed, which is beneficial to the structure strengthening. While the threshold value increased to 1.08 and 3.95 for G‐ and D‐derived structures, respectively, the structures failed due to the bending of tilted struts and exhibited worsened mechanical properties (Figure S5A,C, Supporting Information). For P‐derived structures, even brittle fracture of struts occurred in P‐*t*_3.23_‐[110], showing the minimum peak stress (Figure S5E, Supporting Information). The FE‐simulated stress distributions are in agreement with the experimental structure deformations. The stress gradually concentrated in the struts with increasing threshold values (Figure S5B,D,F, Supporting Information). For these structures in [111] direction, the strength of G‐ and P‐derived structures were weakened with the threshold values increased, but D‐derived structures exhibited better mechanical properties at a threshold value of 1.49 (D‐*t*_1.49_‐[111]) (Figure S6, Supporting Information). As the threshold value increased in the compression process, the deformation mechanism of G‐ and P‐derived structures changed from wall‐stretching‐dominated to strut‐bending‐dominated. In contrast, the D‐derived structures exhibited a mixed deformation mode of bending and stretching dominated due to the presence of vertical struts, which can also be confirmed in the FE simulations (Figure S7, Supporting Information).

**Figure 4:**
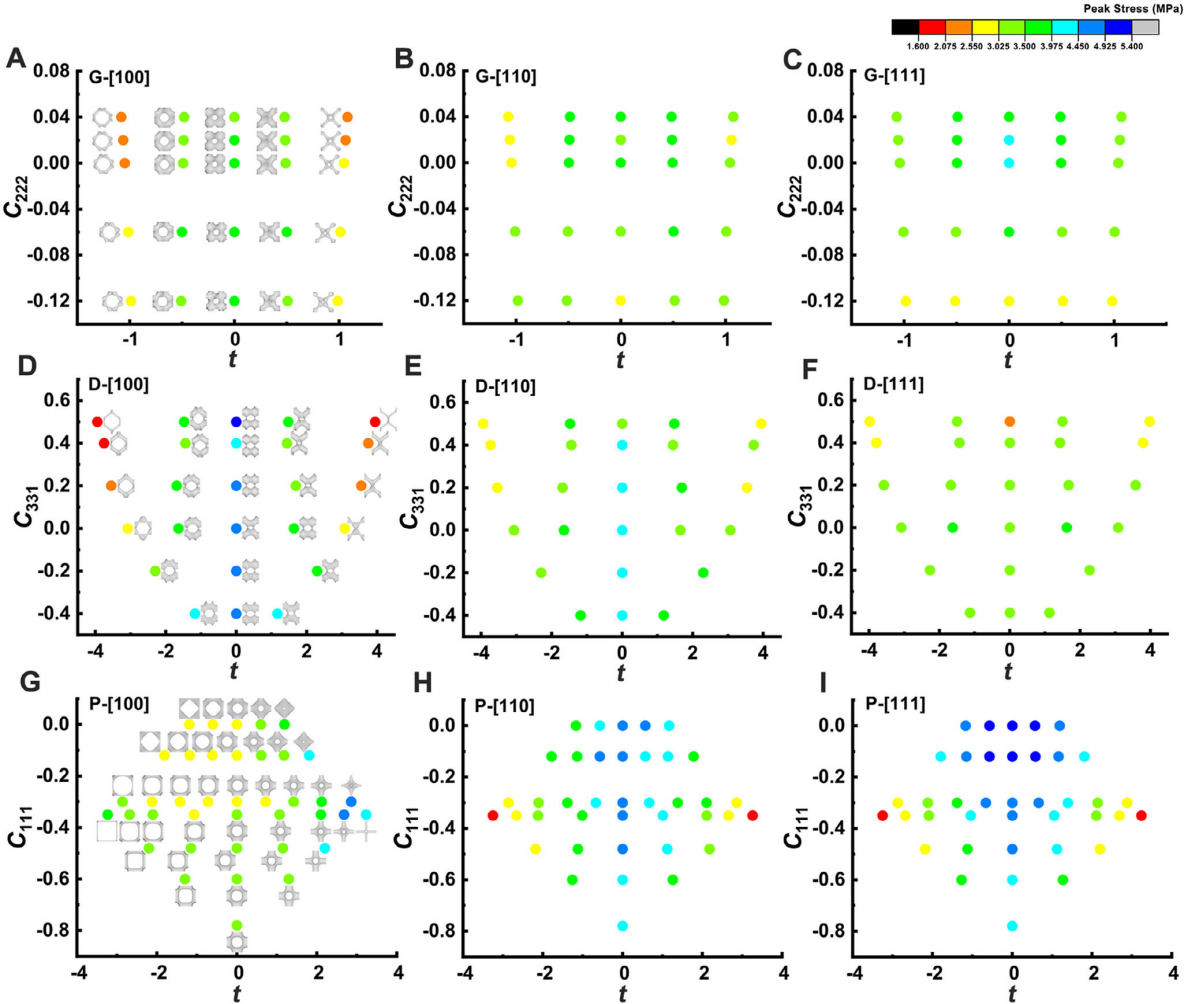
The influence of the parameters *c*_hkl_ and *t* on the geometries and peak strength of G‐, D‐ and P‐derived structures in three characteristic directions. G‐derived structures in A) [100], B) [110], and C) [111] directions. D‐derived structures in D) [100], E) [110], and F) [111] directions. P‐derived structures in G) [100], H) [110], and I) [111] directions.

What's more, we constructed a series of G‐, D‐ and P‐derived structures incorporating various combinations of high‐order Fourier coefficients *c*_hkl_ and threshold values *t*, including three characteristic orientations. The influence of the two parameters (*c*_hkl_ and *t*) on structural geometries and peak strength is shown in **Figure** **4**. It is worth noting that *t* represents the average value of the threshold range spanning the two boundaries. The G‐derived structures exhibited minimal mechanical anisotropy due to their intrinsic homogeneous structural characteristics, suggesting their mechanical properties were less affected by the changes in structural parameters. In contrast, D‐derived structures displayed certain anisotropy, with mechanical properties deteriorated as the threshold value increased, particularly for the [100] orientation, which was most sensitive to variations in *t*. P‐derived structures, on the other hand, exhibited higher strength overall; however, they also demonstrated notable mechanical anisotropy. Specifically, the mechanical properties of P‐derived structures in the [100] direction improved as the threshold value increased, while those in the [110] and [111] directions showed the opposite trend. In general, the wide range of mechanical properties observed across these structures provides a valuable guideline for selecting suitable structures tailored to specific applications.

#### 2.2.3 Optimization of Structural Mechanics by Fine Controlling of Surface Curvature

It is worth noting that, Fourier synthesis allows precise control over hyperbolic surface structures, enabling the design of surfaces with specific node or strut sizes. As shown in Figure S8 (Supporting Information), the curvature of G‐, D‐, and P‐derive surfaces could be adjusted to achieve specified structural features, with variations depending on the topological configuration. In Figure S9 (Supporting Information), we demonstrate how increasing node sizes (blue arrow path) or strut diameters (red arrow path) in three directions ([100], [110], and [111]) affects structural mechanics. Notably, the [110] and [111] orientations of the sample P‐node5 were not fabricated due to excessive overhangs during fabrication. The parameters for these structures are provided in Tables S3–S5 (Supporting Information). Compression test results (Figure S10, Supporting Information) revealed that peak stress generally increased with larger node sizes across G‐, D‐, and P‐derived structures, except for the [100] orientation of P‐derived structures. When strut diameters were increased while keeping nodes unchanged, the compressive strength of certain structures decreased, e.g., [100] direction of P‐derived and [111] direction of G‐derived structures, whereas others exhibited improved mechanical performance.

**Figure 5:**
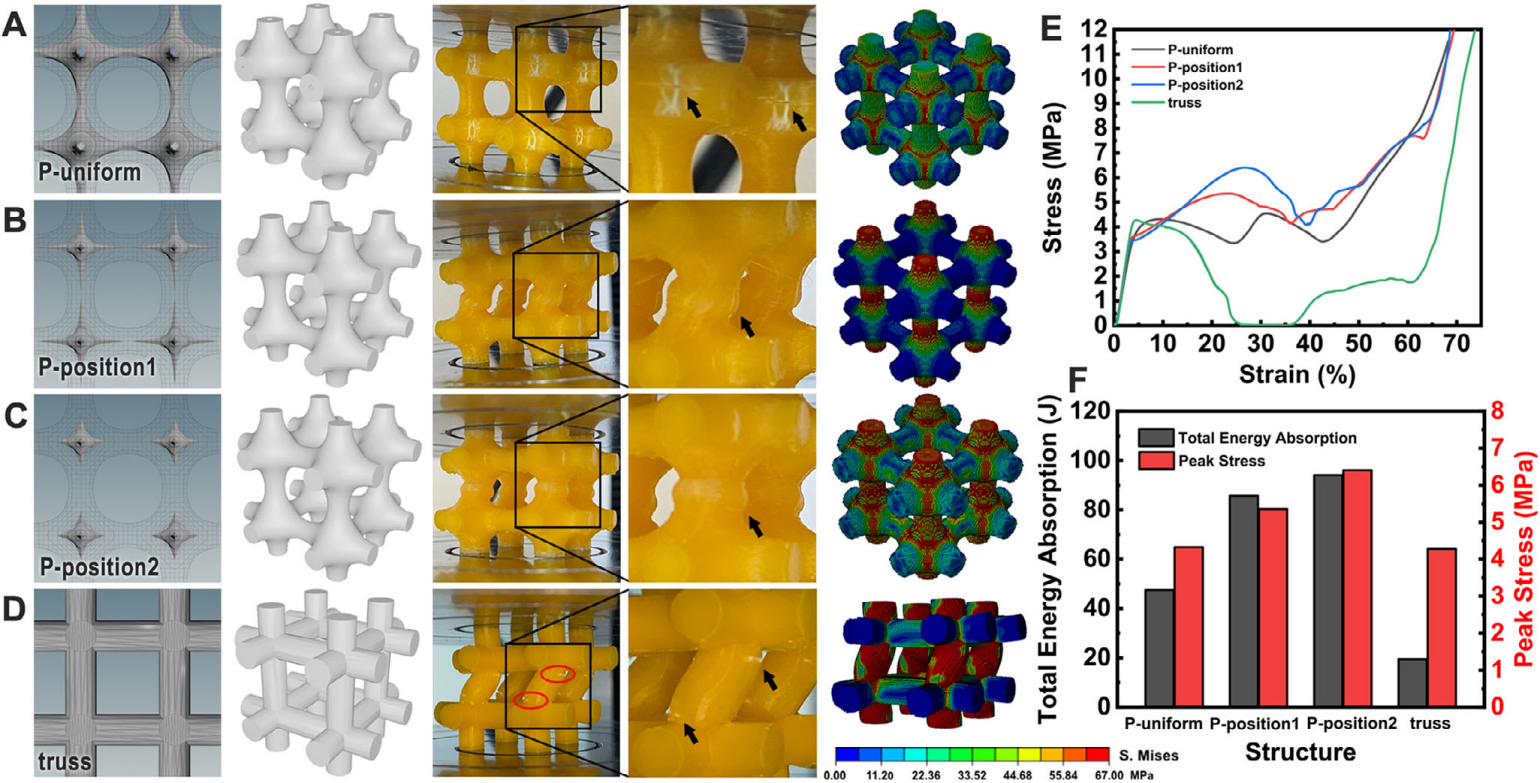
Structural mechanics optimization through the regulation of surface curvature and position based on Fourier synthesis. A) Model and deformation mechanism of P structure with uniform wall thickness, the solid and shaded parts represent the inner and outer walls of the structure, respectively. B) Model and deformation mechanism of optimized P structure with the nodes relatively thickened. C) Model and deformation mechanism of optimized P structure with the struts relatively thickened. D) Model and deformation mechanism of the traditional truss structure. E) stress‐strain curves of these structures. F) The values of peak stress and total energy absorption extracted from the stress‐strain curves. The black arrows in the enlarged images indicate where the deformation occurs.

As we employed the Fourier synthesis method incorporating higher‐order terms to construct the corresponding hyperbolic surface structures, we are able to systematically adjust the wall thickness at any geometrical positions in the structure. Mechanically optimized structures with controllable non‐uniform wall thickness can be achieved by designing inner and outer surfaces with varying shape and curvature settings to improve stress distributions, which overcomes the limitations inherent in traditional simple‐level surfaces. For instance, in the case of P‐surface structures, the traditional approach involves setting two different threshold values for a single‐level surface equation to produce uniform wall thickness (P‐uniform structure, **Figure** **5**A). However, compression testing and FE simulations reveal that stress concentration occurs at nodes, leading to structural failure.

To address this issue, the structure can be optimized under constant mass by designing the non‐uniform wall thickness according to stress distribution. The improvement of the structural mechanics was achieved by increasing the wall thickness at nodal sites. As shown in Figure 5B, thickening node regions (P‐position1) caused failure to shift from nodal collapse to strut buckling, which was also confirmed in FE analysis that maximum stress appeared in struts. The most effective optimization was achieved when both nodes and struts failed simultaneously, as demonstrated by P‐position2 (Figure 5C). Compression tests and FE simulations confirmed that this optimized structure exhibits synchronized cracking at nodes and struts. Additionally, in comparison to traditional truss structures (Figure 5D), which fail rapidly due to stress concentration at sharp corners of nodes, hyperbolic surfaces demonstrate superior load dispersion and mechanical resistance. Stress‐strain curves for all tested structures are shown in Figure 5E, and the optimized P‐position2 structure exhibited the highest compressive strength. The comparison of stress‐strain curves between experimental and FE simulations is shown in Figure S11 (Supporting Information). Although truss structures showed high stiffness, their poor load‐carrying capacity and energy absorption resulted in failure at a strain of 26%. Analysis of stress‐strain curves (Figure 5F) indicate that peak stress and energy absorption are significantly improved in the hyperbolic surface structures through curvature and positional adjustments. Compared to the traditional truss structure, the optimized P‐position2 structure achieved a 49.80% and 383.69% increase in peak stress and energy absorption, respectively. Compared to the original P‐uniform structure, P‐position2 structure exhibited a 48.10% increase in peak stress and 98.02% improvement in energy absorption. The specific parameters of P‐uniform and P‐optimized structures are shown in Table S6 (Supporting Information). These results highlight the effectiveness of this optimization approach and the structures can be flexibly designed according to the actual requirements.

### 2.3 Applications of TPHSs and the Structural Assembly by Mortise‐Tenon Joints

Although TPHSs exhibit significant advantages in mechanical properties, their application in large‐scale engineering construction remains a formidable challenge. These hyperbolic geometric structures cannot be disassembled and reassembled in modular fashion like LEGO bricks. Even if the curved surfaces are disassembled, it is impossible to preserve surface continuity while maintaining their original mechanical properties. Therefore, TPHSs could only be manufactured as integral components via 3D printing technology, with their length scale severely limited by printer size and high costs.

Based on the above findings, the structural features and mechanical properties of TPHSs can be effectively controlled through Fourier construction. By employing higher threshold values and appropriate Fourier coefficients, the structure transformed from a layered hyperbolic surface to a rod‐like configuration, enabling the decomposition and reassembly of basic structural units. Consequently, we proposed a strategy for disassembling and reassembling rod‐like hyperbolic structures using mortise‐tenon joints.

**Figure 6:**
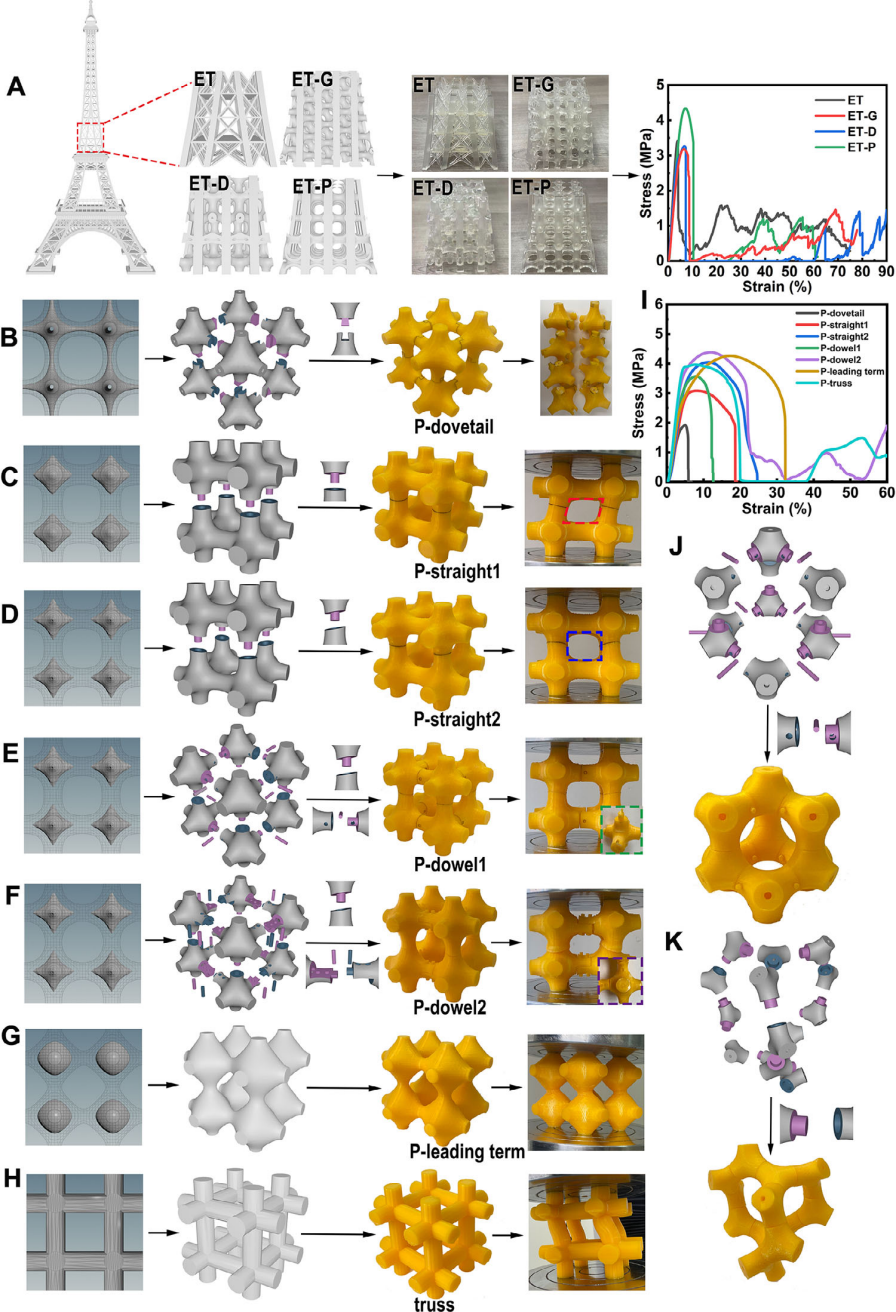
The applications and disassembly‐assembly of hyperbolic surface structures. A) Models and mechanical properties of the Eiffel Tower structure and rod‐like G, D, P infilled structures with the same framework. B) Models and deformation mechanism of P‐assembly structure connected by dovetail joints. C) Models and deformation mechanism of P‐assembly structure connected by straight‐tenon in the vertical direction. D) Models and deformation mechanism of P‐assembly structure connected by beveled‐straight‐tenon in the vertical direction. E) Models and deformation mechanism of P‐assembly structure connected by beveled‐straight‐tenon in the vertical direction and single pin‐tenon in the horizontal direction. F) Models and deformation mechanism of P‐assembly structure connected by beveled‐straight‐tenon in the vertical direction and double‐pin‐tenon in the horizontal direction. G) Integrally printed leading‐term approximate P structure. H) Integrally printed traditional truss structure. I) Stress‐strain curves of these structures. J) Assembled G structures. K) Assembled D structures.

First, we compared the rod‐like G, D, and P structures with the truss structure of the middle section of the Eiffel Tower under load (**Figure** **6**A). We designed structures featuring identical external frames but incorporating truss and rod‐like G, D, and P infill configurations, respectively. The specific parameters of rod‐like G, D, and P structures are listed in Table S7 (Supporting Information), which were chosen according to the mechanical properties as shown in Figure 4. Due to the thinness of the trusses of the Eiffel Tower structure and the resolution limitations of FDM 3D printing, Stereolithography Appearance (SLA) 3D printing was used for manufacturing these structures. From the stress‐strain curves, it is evident that the structure infilled with a rod‐like P configuration exhibited significantly greater strength compared to the original Eiffel Tower truss structure. While the rod‐like G and D configurations were slightly weaker in terms of strength, they demonstrated nearly doubled energy absorption capacity, suggesting the excellent mechanical properties of rod‐like hyperbolic surface structures.

Mortise‐tenon joints from traditional Chinese architecture and furniture exhibit a variety of constructions and possess exceptional mechanical properties, offering valuable insights for structural connections.^[^ ^26^ ^]^ These joints are semi‐rigid and connected based on the principle of concave‐convex complementarity,^[^ ^27^ ^]^ which are capable of transferring vertical loads effectively and withstand horizontal stress as well as bending moments. Furthermore, due to the friction between components, these joints demonstrate significant damping and energy absorption capacities.^[^ ^27b,c^ ^]^ Due to limitations in current structural design methods and FDM printing resolution, it is challenging to assemble complex multi‐layer mortise‐tenon assemblies; therefore, we also opted for a two‐layer design. A common dovetail joint was selected to demonstrate the assembly of the rod‐like P structure (P‐ dovetail), however, this structure collapsed quickly under compressive load as shown in Figure 6B. The compression tests conducted on the assembled joints with only vertically or horizontally connection revealed that the dovetail joint is not suitable for supporting the load resistance required for the rod‐like P structure, particularly in the horizontal direction. The joint barely resists transverse stretching, leading to structural collapse under low strain (Figure S12, Supporting Information). Therefore, it is necessary to design appropriate mortise‐tenon joints for the vertical and horizontal directions respectively.

For vertical connections, we designed straight‐tenons with large wrapping areas (Figure 6C). Since the horizontal part was printed as an integrated component, the P structure connected by straight‐tenons (P‐straight1) experienced significant strain, but obvious shear forces occurred during compression, leading to structural collapse (the deformation hole is marked by the red dashed box in Figure 6C). To address this issue, we designed a beveled straight‐tenon (P‐straight2) with a 15° inclination relative to the horizontal direction (Figure 6D). This design effectively counteracted the negative impact of shear forces on the structure, as evidenced by the deformation hole changing shape to a rectangle (marked by the blue dashed box in Figure 6D). On the other hand, to establish stable horizontal connections, we designed a dowel pin tenon for P‐straight2 with stronger interacting tensile force in the horizontal direction (P‐dowel1). This design significantly enhanced the mechanical performance of the assembled rod‐like P structure compared to the dovetail joint (Figure 6E). However, upon observing the collapsed fragments (marked by the green dashed box in Figure 6E), we found that the dowel pins were too thin, and the direction of the pin‐holes was parallel to the 3D printing axis, resulting in weak connections. Therefore, we improved our design with a vertical double‐dowel‐pin assembly for the P structure (P‐dowel2) by adjusting the direction and thickness of the pins (Figure 6F). The compression process and fragments showing good horizontal connections (marked by the purple dashed box in Figure 6F) demonstrate that the vertical double‐dowel‐pin joints effectively resisted transverse stretching, enhancing both structural integrity and mechanical performance under compressive load.

For comparison, we fabricated and tested the leading‐term approximate rod‐like P structure and the traditional truss structure (Figure 6G,H). The stress‐strain curves (Figure 6I) demonstrated that the assembled P‐dowel2 structure exhibited superior performance, with a peak stress 10% higher than that of the integrally printed truss structure. Furthermore, its performance even surpassed that of the level‐set leading‐term rod‐like P structure. We attribute this enhanced mechanical performance to the gaps within the mortise‐tenon joints, which allow the structure to dissipate stress and absorb energy through small deformations when subjected to external loads. Notably, we applied the structural optimization principle described in section 2.2.2 to design the surface curvature and position of the P structures. Specifically, this principle ensures that when nodes and struts collapse almost simultaneously under load, the structure achieves its strongest mechanical performance. The parameters of the level‐set surfaces for the assembled P structures are provided in Table S8 (Supporting Information). Additionally, our mortise‐tenon joint design is not limited to rod‐like P structures but can also be applied to other rod‐like structures such as G and D configurations (Figure 6J,K). For these structures, we designed pin‐tenon joints for the rod‐like D structure and straight‐tenon joints for the rod‐like G structure to enable structural assembly. The mortise‐tenon joint concept can be extended to other hyperbolic surface structures with different joint designs and has potential applications in prefabricated engineering.

In our current study, we have not yet thoroughly explored the issues of fatigue failure and tolerance accumulation; however, these aspects are integral considerations in our structural design. The mortise‐tenon structure is designed with interlocking geometries that distribute external forces over larger contact areas, thereby minimizing stress concentrations at individual points and potentially delaying fatigue failure. Additionally, we incorporated redundant design elements by introducing multiple supportive connection points at critical locations. This allows for the compensation or distribution of tolerances at a single node among others, reducing the risk of cumulative tolerance issues. Further investigation is underway to explore these aspects, particularly focusing on long‐term durability and assembly precision.

## 3 Conclusion

In this paper, a universal method inspired by crystallographic Fourier synthesis is proposed for the design of TPHS structures, which significantly broadens the design space for novel TPHSs. By superimposing the Fourier components associated with different reflections within space group symmetries, various innovative hyperbolic surface structures with diverse mechanical characteristics have been developed, where the strongest structure achieved a peak stress exceeding that of the conventional strongest P surface structure by 86.21% under the same mass. Furthermore, the mechanical performance of TPHS structures can be optimized by adjusting surface curvature and position to create non‐uniform structures that align with stress distribution patterns. This approach leads to significant improvements in their mechanical properties, resulting in a 98.02% increase in energy absorption and a 48.10% rise in peak stress compared to the original structure. Additionally, this study demonstrates the disassembly and assembly of TPHS structures through mortise‐tenon joints connecting rod‐type components. The assembled P structure exhibited a 10.54% higher peak stress compared to traditionally truss structures, showing the potential of modular construction techniques for enhancing structural performance. This work introduces new possibilities for the development of advanced materials and lightweight structures with tailored mechanical behaviors. This approach could pave the way for innovative applications in various fields, such as aerospace, construction, and robotics, where high‐performance, adaptable, and energy‐efficient structures are highly desired. The integration of modular assembly techniques further highlights the potential for scalable manufacturing and adaptive design strategies in future TPHS applications.

## 4 Experimental Section

### Scaffolds Design and Structural Modeling

G‐, D‐ and P‐related hyperbolic surfaces can be approximated by the level surface equations in terms of the Fourier series using the structure factor *F*(***k***) with a given reciprocal lattice vector ***k*** and the phase shift α(***k***) (see Equation (1)). Approximations can be obtained by truncating the series to the leading term, giving the G‐, D‐ and P‐related hyperbolic surfaces by simple expressions. In addition, more accurate G‐, D‐ and P‐derived structures can be constructed by introducing higher‐order Fourier terms, where the coefficient indicates the intensity of corresponding reflections.^[^ ^25^ ^]^ The detailed higher‐order Fourier terms of G, D, and P, are shown in Tables S9–S11 (Supporting Information). By setting different coefficients *c*_hkl_ and threshold values *t*, the curvature of the hyperbolic surface can be adjusted and even novel structures can be constructed. All structures were generated using Houdini, a software that produced complex 3D geometries with the use of implicit functions. Models in section 2.2.1 are 30 mm × 30 mm × 30 mm in size with 2 × 2 × 2 unit cells and volume fractions of 23.1% ± 0.5%. Models in section 2.2.2 are 30 mm × 30 mm × 30 mm with unit cells of 3 × 3 × 3 and volume fractions of 19.7% ± 0.5%. Models in section 2.2.3 are 30 mm × 30 mm × 30 mm with unit cells of 2 × 2 × 2 and volume fractions of 19.8%. Models in section 2.3 are 85 mm × 85 mm × 85 mm with unit cells of 2 × 2 × 2 and volume fractions of 19.0%.

### Additive Manufacturing Process

All models were manufactured by FDM 3D printer (Raise 3D Pro2) with polylactic acid (PLA) filament (1.75 mm) except the “Eiffel Tower structures,” which were printed by SLA technology (Formlabs Form 3) for the limitation of the resolution, using the transparent photosensitive resin. Both printing materials were obtained from the Polymaker components. Some main printing parameters of FDM were given as follows: layer thickness of 0.15 mm, printing speed of 40 mm s^−1^, nozzle diameter of 0.3 mm, printing temperature of 205 °C and build plate temperature of 60 °C. As for SLA: layer thickness of 0.1 mm, laser spot size of 85 µm, laser wavelength of 405 nm, laser power of 250 mW, and post‐processing of washed in isopropyl alcohol (IPA) for 10 min and cured at 60 °C for 10 min.

### Mechanical Compressive Test

To obtain the mechanical properties of these structures, compressive tests were performed using FR‐103C testing machine, and TM2101 software to control the machine and record the measurements. Since 3D printed scaffolds were highly anisotropic, all the samples were placed between two flat hard metal machine platens at the printing direction, so that the loading direction was the printing direction. Mechanical compressive tests were implemented with a 50 kN load cell, and a deformation rate of 1 mm min^−1^, when densification occurred, the test was terminated. Stress‐strain curves were accordingly extracted considering square cross‐section area for calculating stress which refers to the whole rectangular cross‐sectional area of the scaffold comprising both scaffold material and voids. Elastic modulus and compressive strength were calculated according to the slope of the first linear region in stress‐strain curves and the highest recorded stress, respectively. The mechanical properties of the base material were obtained from the uniaxial compressive test on a PLA cylinder sample with a size of Φ12.7 mm × 25.4 mm with a loading speed of 1 mm min^−1^ according to ASTM D695, giving an elastic modulus of 1400 MPa and yield stress of 57 MPa. Specimens were fabricated by FDM using the same printing parameters as TPHS structures in this study.

### FE Simulation Procedure

Through the in‐house meshing software written in C++, the mesh was automatically generated from the parameters of each structure by the voxel method and imported into Abaqus 6.14 for FE calculation. The mechanical properties of the materials used in the simulation were measured experimentally, with an elastic modulus of ≈1400 MPa, a Poisson's ratio of ≈0.3, and a yield strength of ≈57 MPa, and the strain hardening phenomenon was considered. The type of analysis performed was “Explicit, dynamic.” The element type was C3D8R with a size of 0.25 mm. In the simulation of each structure, the upper and lower end faces were constrained by rigid bodies to simulate the action of the compression plate in the experiment. The contact was considered between the rigid body and the structure as well as the structure itself. Therefore, the general contact was imposed on the simulation model. In the contact property, the tangential behavior adopts a penalty friction formulation with a friction coefficient of 0.3, and the normal behavior was defined as hard contact. In all simulations, fix the lower rigid body and impose displacement boundary conditions on the upper rigid body to be consistent with the actual situation of the compression experiments. The loading speed of the upper rigid body is 1 mm s^−1^. The deformation and Von Mises stress contours of all structures were exported as simulation results.

## Conflict of Interest

The authors declare no conflict of interest.

## Author Contributions

L.H. proposed the concept and supervised the project. W.Y. supervised the project. Y.Z performed the experiments, analyzed the data, and wrote the draft of the manuscript. Y.Z. and J.Z. performed the FE simulation. Y.L. and S.C. reviewed the manuscript and participated in the discussion. J.Z., W.Y, and L.H. reviewed and edited the manuscript.

## Supporting information



Supporting Information

Supplemental Movie 1

Supplemental Movie 2

Supplemental Movie 3

Supplemental Movie 4

## Data Availability

The data that support the findings of this study are available from the corresponding author upon reasonable request.
