# Supplementary material for: Multi‐Mechanical Regulation of 3D Printed Triply Periodic Hyperbolic Surfaces via Fourier Synthesis‐Based Free Modeling
Source: Adv Sci (Weinh). 2025 May 19;12(29):70028. doi: 10.1002/advs.202503694 (PMC12362832; doi:10.1002/advs.202503694)
Supplement: Supplementary file 1 — Supporting Information [file ADVS-12-70028-s001.pdf]

ADVANCED SCIENCE
Open Access

Supporting Information

for *Adv. Sci.*, DOI 10.1002/advs.202503694

Multi-Mechanical Regulation of 3D Printed Triply Periodic Hyperbolic Surfaces via Fourier Synthesis-Based Free Modeling

Yanhong Zhang, Junming Zhang, Zhimei Zhou, Yan Li, Shunai Che, Weidong Yang and Lu Han**

Supporting Information

Multi-Mechanical Regulation of 3D Printed Triply Periodic Hyperbolic Surfaces via Fourier Synthesis-Based Free Modeling

Yanhong Zhang,[†] *Junming Zhang,*[†] *Zhimei Zhou, Yan Li, Shunai Che, Weidong Yang* and Lu Han**

Dr. Y. Zhang, Z. Zhou, Prof. S. Che, Prof. L. Han
School of Chemical Science and Engineering, Tongji University, Shanghai, 200092, China.
E-mail: luhan@tongji.edu.cn

J. Zhang, Prof. Y. Li, Prof. W. Yang
School of Aerospace Engineering and Applied Mechanics, Tongji University, Shanghai, 200092, China.
E-mail: yangwd@tongji.edu.cn

Prof. S. Che
School of Chemistry and Chemical Engineering, State Key Laboratory of Composite Materials, Shanghai Key Laboratory for Molecular Engineering of Chiral Drugs, Shanghai Jiao Tong University, Shanghai, 200240, China.

† These authors contributed equally.

Movie S1. The variation of hyperbolic surface with the second-order Fourier term added under the space group symmetry of $Pm\bar{3}m$.

Movie S2. The variation of hyperbolic surface with the third-order Fourier term added under the space group symmetry of $Pm\bar{3}m$.

Movie S3. The variation of hyperbolic surface with the fourth-order Fourier term added under the space group symmetry of $Pm\bar{3}m$.

Movie S4. The variation of hyperbolic surface with the fifth-order Fourier term added under the space group symmetry of $Pm\bar{3}m$.

Equation S1. Calculation equation for the P surface, c is the coefficient for corresponding hkl Bragg reflections. The coefficients listed in the table represent the Fourier coefficients used to fit triply periodic minimal surfaces (TPMS).[1]

$$\{h\ k\ l\} = \sum_{hkl} c\{\cos(hx)[\cos(ky)\cos(lz) + \cos(ly)\cos(kz)] + \cos(hy)[\cos(kz)\cos(lx) + \cos(lz)\cos(kx)] + \cos(hy)[\cos(kz)\cos(lx) + \cos(lz)\cos(kx)]\}$$

h	k	l	Fitting coefficient
Fitting 5 terms			
1	0	0	1
1	1	1	-0.00956335
2	1	0	0.00959441
3	0	0	-0.0181364
2	2	1	-0.0292538
Fitting 2 terms			
1	0	0	1
1	1	1	-0.150736

Equation S2. Calculation equation for the G surface, c is the coefficient for corresponding hkl Bragg reflections. The coefficients listed in the table represent the Fourier coefficients used to fit TPMS.[1]

$$\{h\ k\ l\} = \sum_{hkl}\Bigg[\cos\left(\frac{h+k+l}{4}\right)\Bigg(\Bigg(\cos\left(hx+\frac{l}{4}\right)\cos\left(ky+\frac{h}{4}\right)\cos\left(lz+\frac{k}{4}\right)$$
$$+\cos\left(kx+\frac{h}{4}\right)\cos\left(ly+\frac{k}{4}\right)\cos\left(hz+\frac{l}{4}\right)$$
$$+\cos\left(lx+\frac{k}{4}\right)\cos\left(hy+\frac{l}{4}\right)\cos\left(kz+\frac{h}{4}\right)\Bigg)$$
$$+\cos\left(\frac{h+k+l}{4}\right)\Bigg(\cos\left(kx+\frac{l}{4}\right)\cos\left(hy+\frac{k}{4}\right)\cos\left(lz+\frac{h}{4}\right)$$
$$+\cos\left(lx+\frac{h}{4}\right)\cos\left(ky+\frac{l}{4}\right)\cos\left(hz+\frac{k}{4}\right)$$
$$+\cos\left(hx+\frac{k}{4}\right)\cos\left(ly+\frac{h}{4}\right)\cos\left(kz+\frac{l}{4}\right)\Bigg)\Bigg)$$
$$+\cos\left(\frac{h+k+l}{4}\right)\Bigg(\Bigg(\sin\left(hx+\frac{l}{4}\right)\sin\left(ky+\frac{h}{4}\right)\sin\left(lz+\frac{k}{4}\right)$$
$$+\sin\left(kx+\frac{h}{4}\right)\sin\left(ly+\frac{k}{4}\right)\sin\left(hz+\frac{l}{4}\right)$$
$$+\sin\left(lx+\frac{k}{4}\right)\sin\left(hy+\frac{l}{4}\right)\sin\left(kz+\frac{h}{4}\right)\Bigg)$$
$$+\sin\left(\frac{h+k+l}{4}\right)\Bigg(\sin\left(kx+\frac{l}{4}\right)\sin\left(hy+\frac{k}{4}\right)\sin\left(lz+\frac{h}{4}\right)$$
$$+\sin\left(lx+\frac{h}{4}\right)\sin\left(ky+\frac{l}{4}\right)\sin\left(hz+\frac{k}{4}\right)$$
$$+\sin\left(hx+\frac{k}{4}\right)\sin\left(ly+\frac{h}{4}\right)\sin\left(kz+\frac{l}{4}\right)\Bigg)\Bigg)\Bigg]$$

h	k	l	Fitting coefficient
1	1	0	1
2	2	2	-0.00382453
4	1	1	-0.000878436
3	3	0	0.00225091

Equation S3. Calculation equation for the D surface, *c* is the coefficient for corresponding *hkl* Bragg reflections. The coefficients listed in the table represent the Fourier coefficients used to fit TPMS.[1]

$$\{h\ k\ l\} = \sum_{hkl} c\left[\cos\left(lz - \frac{k}{4} + \frac{h}{4}\right)\left(\cos\left(hx + \frac{l}{4} - \frac{k}{4}\right)\cos\left(ky + \frac{h}{4} - \frac{l}{4}\right)\right.\right.$$
$$\left. + \cos\left(hy + \frac{l}{4} - \frac{k}{4}\right)\cos\left(kx + \frac{h}{4} - \frac{l}{4}\right)\right)$$
$$+ \cos\left(lx - \frac{k}{4} + \frac{h}{4}\right)\left(\cos\left(hy + \frac{l}{4} - \frac{k}{4}\right)\cos\left(kz + \frac{h}{4} - \frac{l}{4}\right)\right.$$
$$\left. + \cos\left(hz + \frac{l}{4} - \frac{k}{4}\right)\cos\left(ky + \frac{h}{4} - \frac{l}{4}\right)\right)$$
$$+ \cos\left(ly - \frac{k}{4} + \frac{h}{4}\right)\left(\cos\left(hz + \frac{l}{4} - \frac{k}{4}\right)\cos\left(kx + \frac{h}{4} - \frac{l}{4}\right)\right.$$
$$\left. + \cos\left(hx + \frac{l}{4} - \frac{k}{4}\right)\cos\left(kz + \frac{h}{4} - \frac{l}{4}\right)\right)$$
$$+ \sin\left(lz - \frac{k}{4} + \frac{h}{4}\right)\left(\sin\left(hx + \frac{l}{4} - \frac{k}{4}\right)\sin\left(ky + \frac{h}{4} - \frac{l}{4}\right)\right.$$
$$\left. + \sin\left(hy + \frac{l}{4} - \frac{k}{4}\right)\sin\left(kx + \frac{h}{4} - \frac{l}{4}\right)\right)$$
$$+ \sin\left(lx - \frac{k}{4} + \frac{h}{4}\right)\left(\sin\left(hy + \frac{l}{4} - \frac{k}{4}\right)\sin\left(kz + \frac{h}{4} - \frac{l}{4}\right)\right.$$
$$\left. + \sin\left(hz + \frac{l}{4} - \frac{k}{4}\right)\sin\left(ky + \frac{h}{4} - \frac{l}{4}\right)\right)$$
$$+ \sin\left(ly - \frac{k}{4} + \frac{h}{4}\right)\left(\sin\left(hz + \frac{l}{4} - \frac{k}{4}\right)\sin\left(kx + \frac{h}{4} - \frac{l}{4}\right)\right.$$
$$\left.\left. + \sin\left(hx + \frac{l}{4} - \frac{k}{4}\right)\sin\left(kz + \frac{h}{4} - \frac{l}{4}\right)\right)\right]$$

h	k	l	Fitting coefficient
1	1	1	1
3	3	1	0.0235559
5	1	1	0.00619501

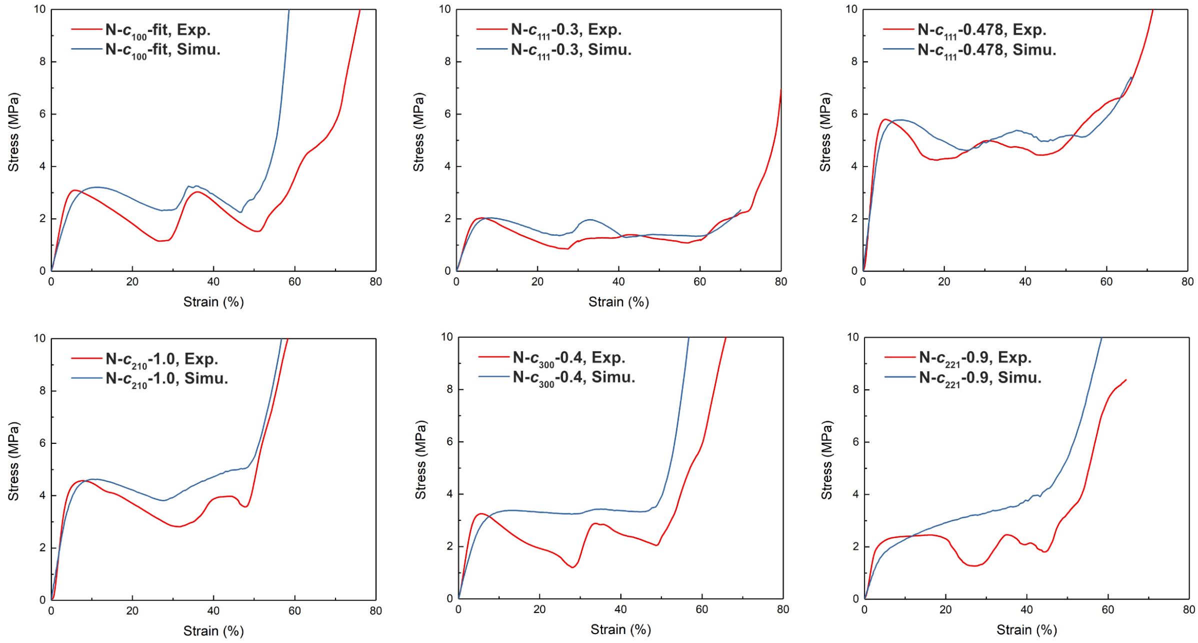

Figure S1. Comparison of stress-strain curves between experimental and FE simulation results for the samples shown in Figure 1.

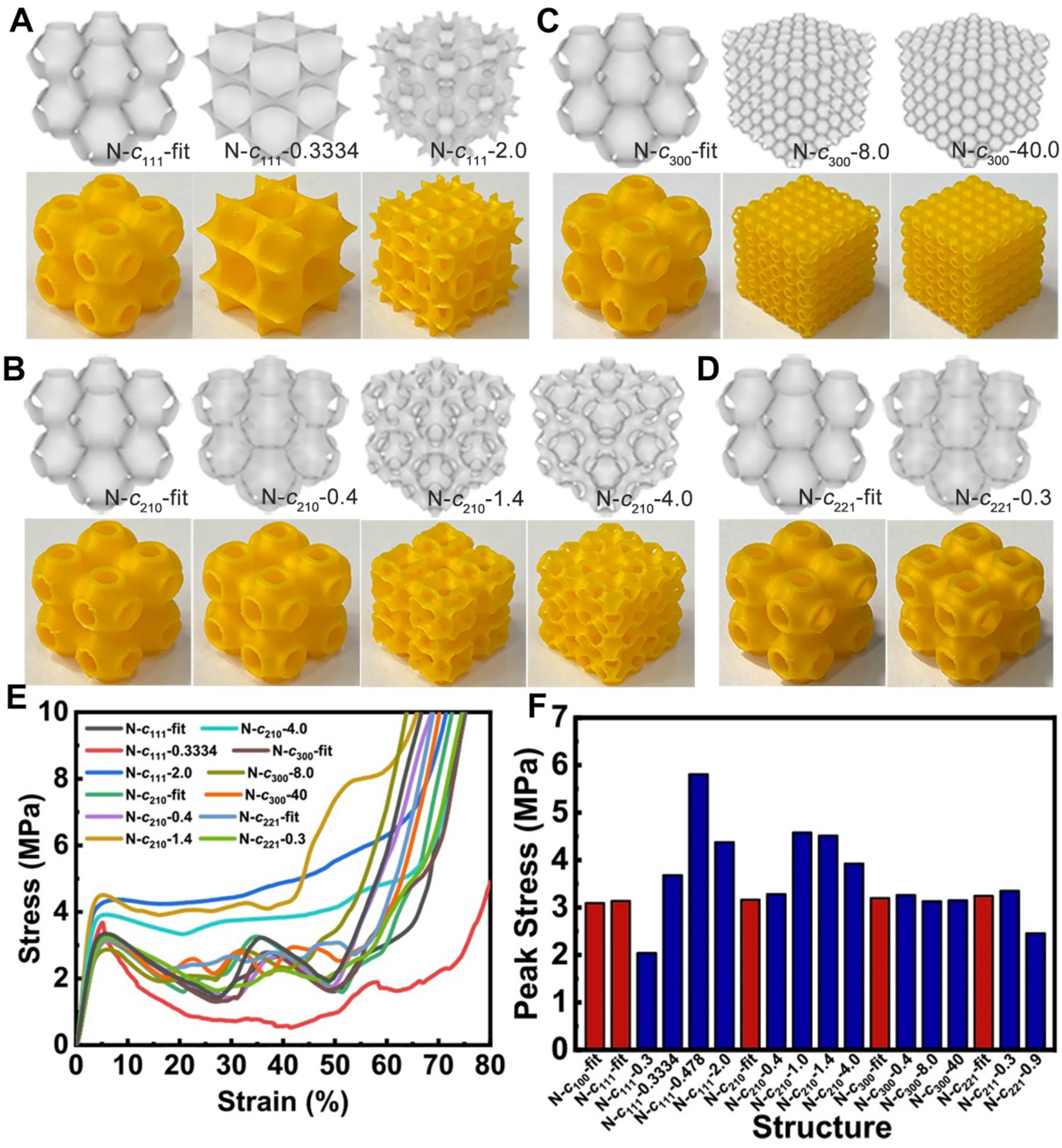

Figure S2. Construction of TPHSs and their mechanical properties. A)-D) hyperbolic surface structures constructed based on P surface with different coefficients of second-, third-, fourth- and fifth-order Fourier terms. E) Stress-strain curves and F) Peak stress of these hyperbolic surface structures.

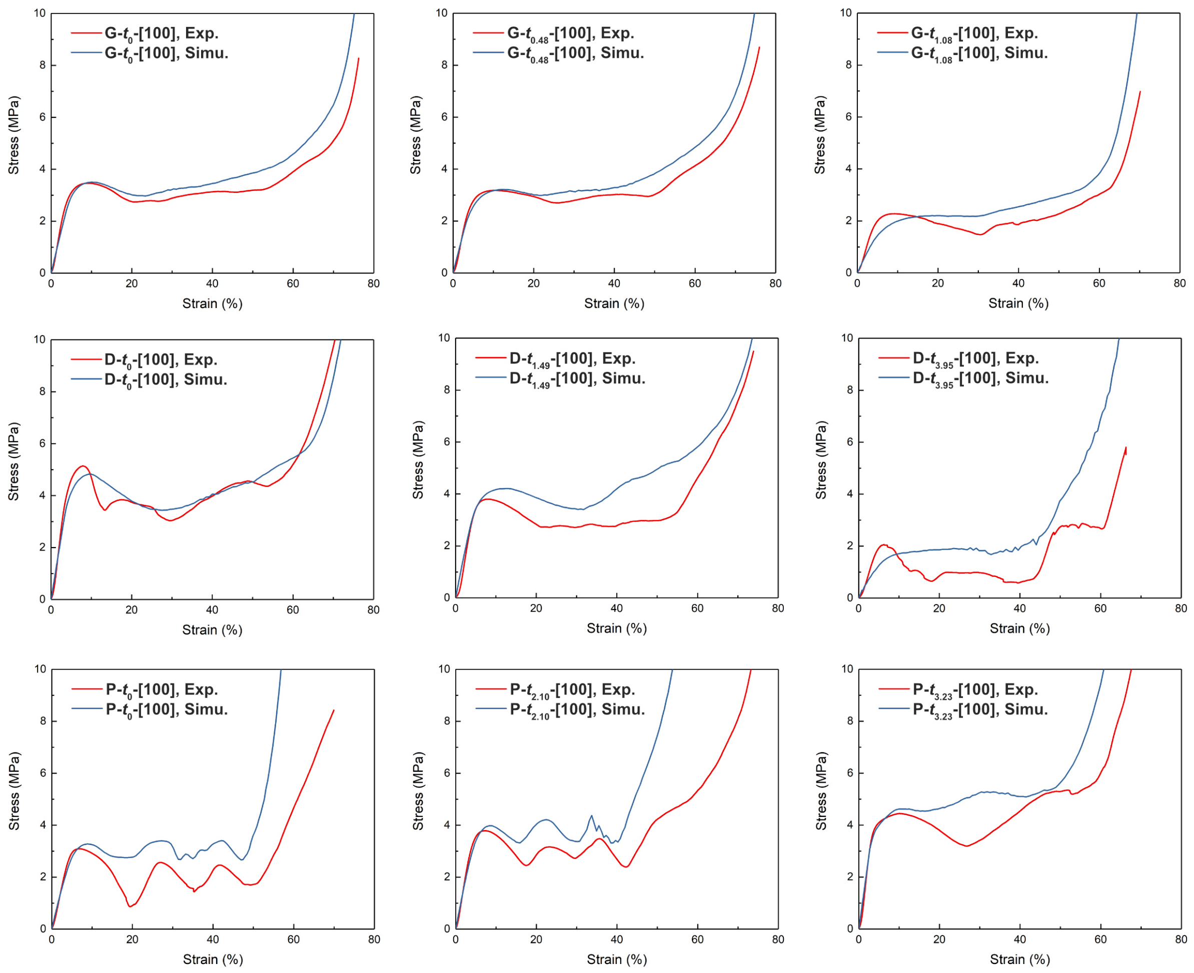

Figure S3. Comparison of stress-strain curves between experimental and FE simulation results for the samples shown in Figure 3.

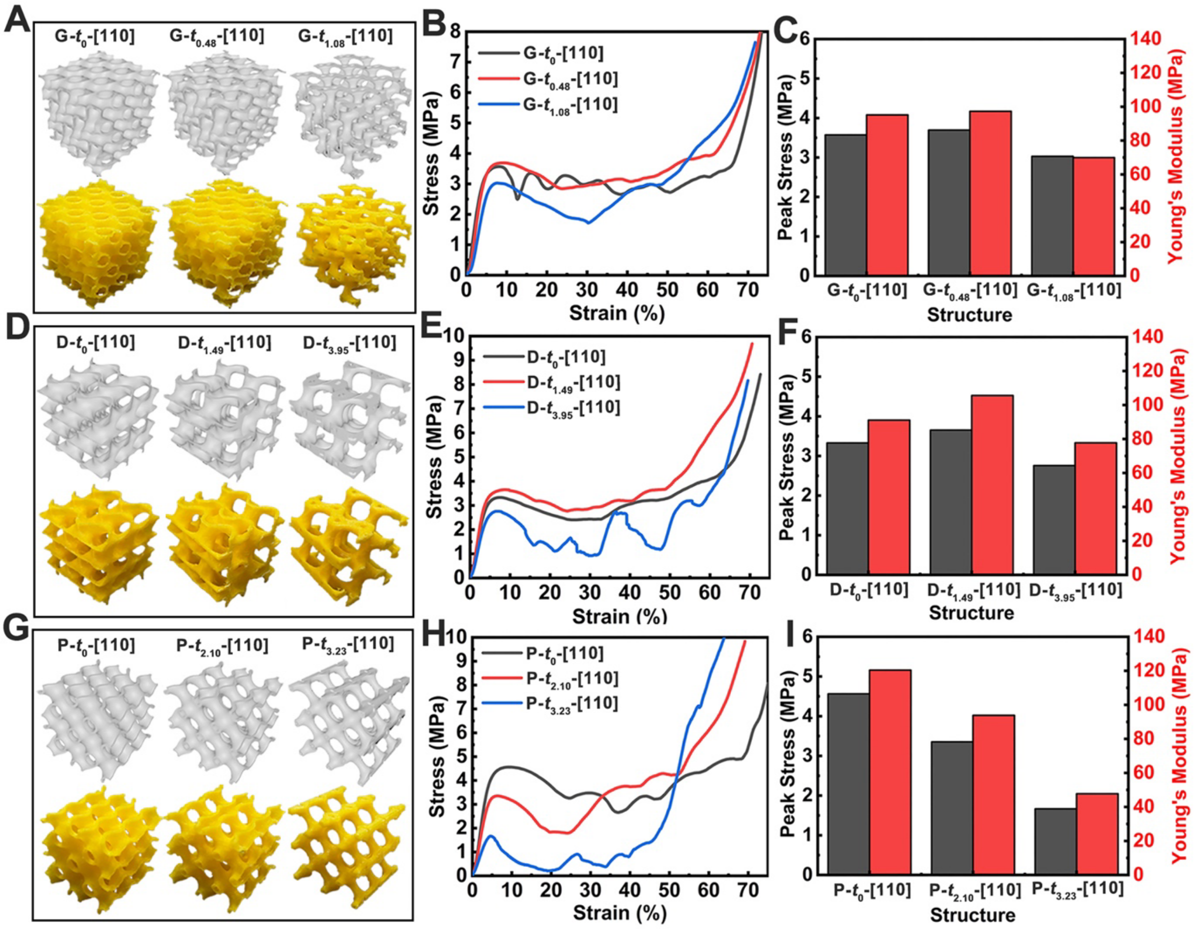

Figure S4. The effect of offset value on the structural mechanical properties in direction [110]. A) CAD and physical models of G-derived structures. B) Stress-strain curves of G-derived structures. C) The values of peak stress and Young's modulus of G-derived structures extracted from the stress-strain curves. D) CAD and physical models of D-derived structures. E) Stress-strain curves of D-derived structures. F) The values of peak stress and Young's modulus of D-derived structures extracted from the stress-strain curves. G) CAD and physical models of P-derived structures. H) Stress-strain curves of P-derived structures. I) The values of peak stress and Young's modulus of P-derived structures extracted from the stress-strain curves.

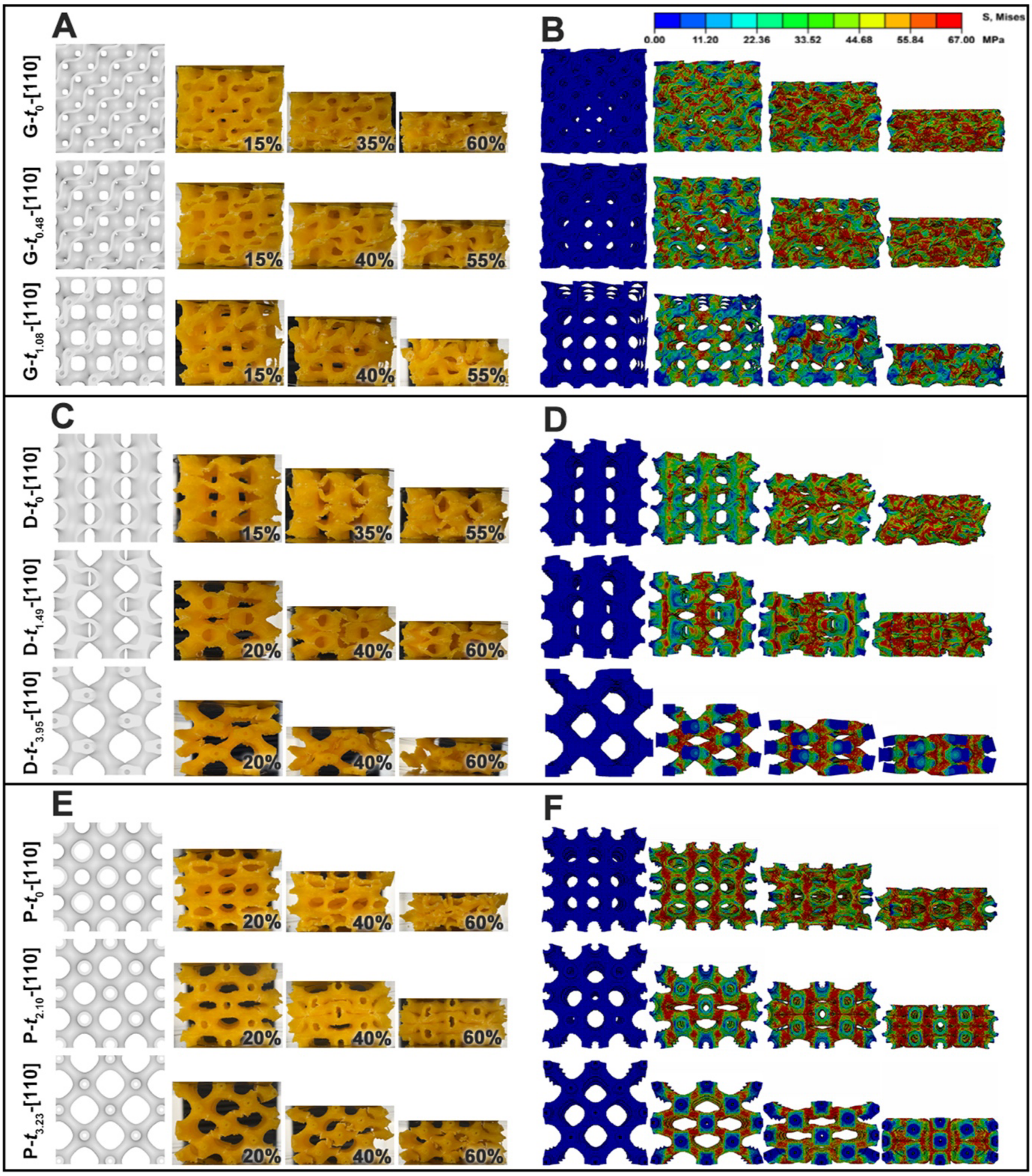

Figure S5. Deformation mechanism of G-, D- and P-derived structures in [110] direction. A) 3D printed G-derived structures under compressive load. B) Corresponding cases from FE simulations. C) 3D printed D-derived structures under compressive load. D) Corresponding cases from FE simulations. E) 3D printed P-derived structures under compressive load. F) Corresponding cases from FE simulations. The colors indicate the local level of stress (von Mises stress).

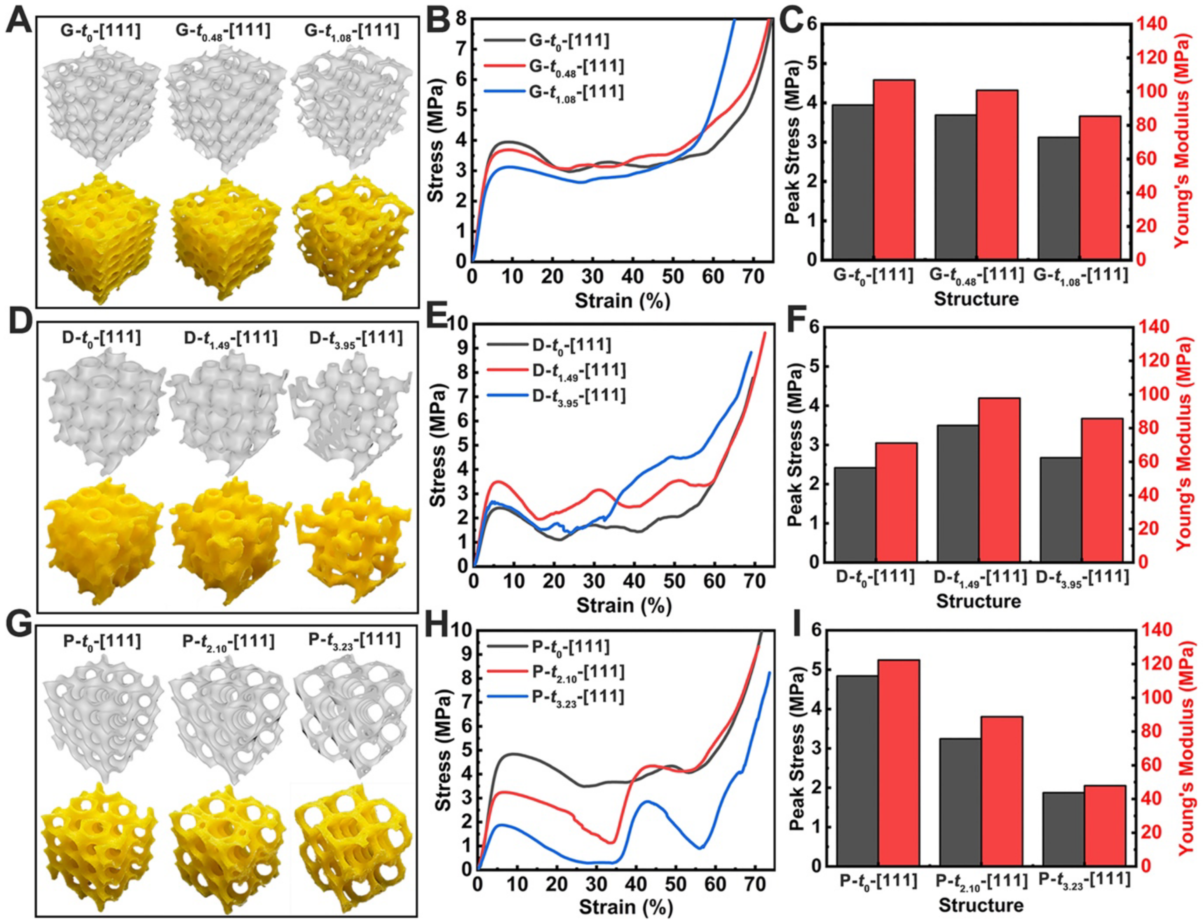

Figure S6. The effect of offset value on the structural mechanical properties in [111] direction. A) CAD and physical models of G-derived structures. B) Stress-strain curves of G-derived structures. C) The values of peak stress and Young's modulus of G-derived structures extracted from the stress-strain curves. D) CAD and physical models of D-derived structures. E) Stress-strain curves of D-derived structures. F) The values of peak stress and Young's modulus of D-derived structures extracted from the stress-strain curves. G) CAD and physical models of P-derived structures. H) Stress-strain curves of P-derived structures. I) The values of peak stress and Young's modulus of P-derived structures extracted from the stress-strain curves.

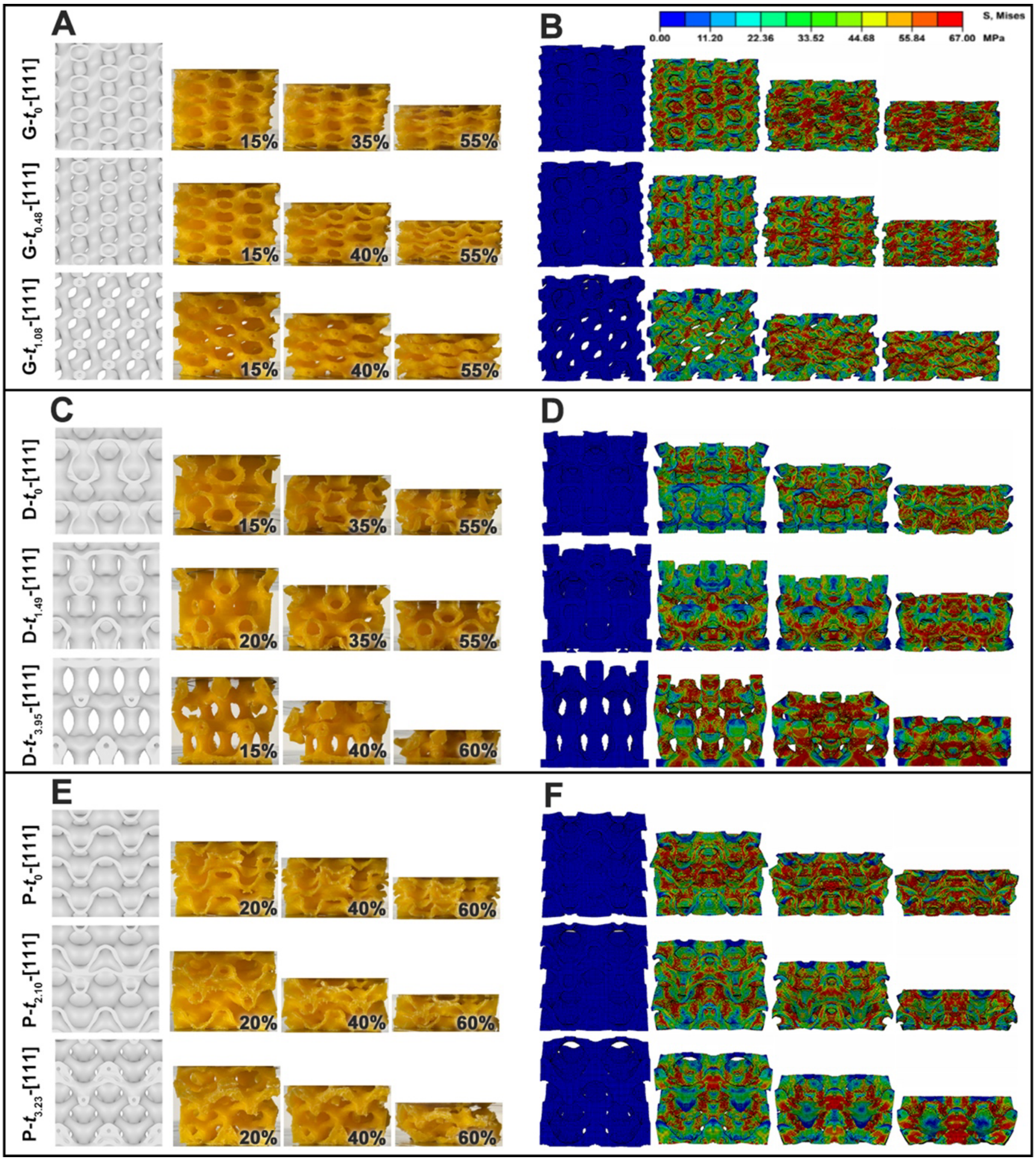

Figure S7. Deformation mechanism of G-, D- and P-related structures in [111] direction. A) 3D printed G-derived structures under compressive load. B) Corresponding cases from FE simulations. C) 3D printed D-derived structures under compressive load. D) Corresponding cases from FE simulations. E) 3D printed P-derived structures under compressive load. F) Corresponding cases from FE simulations. The colors indicate the local level of stress (von Mises stress).

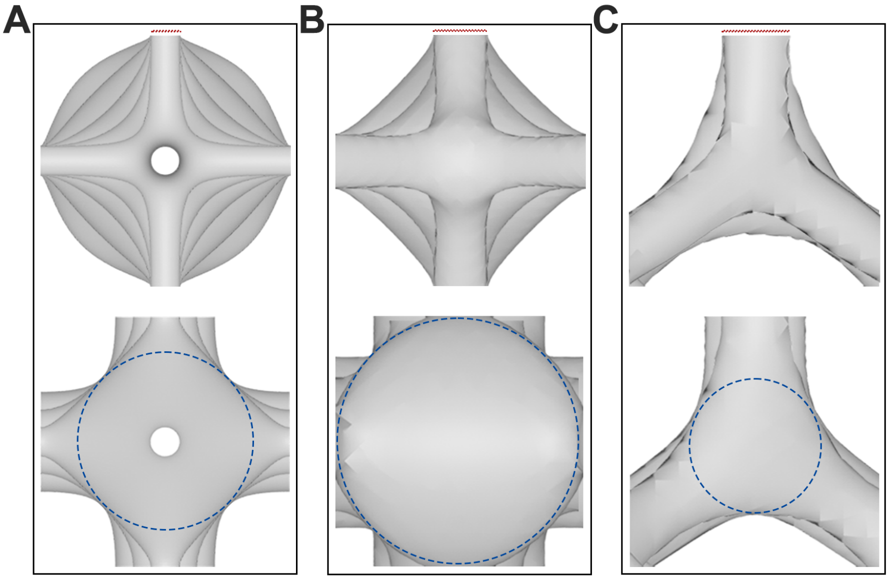

Figure S8. Regulation of hyperbolic surface curvature. the regulation of node under the same strut (top) and the regulation of strut under the same node (bottom) of A) P, B) D and C) G structure. The red dotted line on the top indicates the diameter of the rod and the blue dotted circle indicates the size of the node.

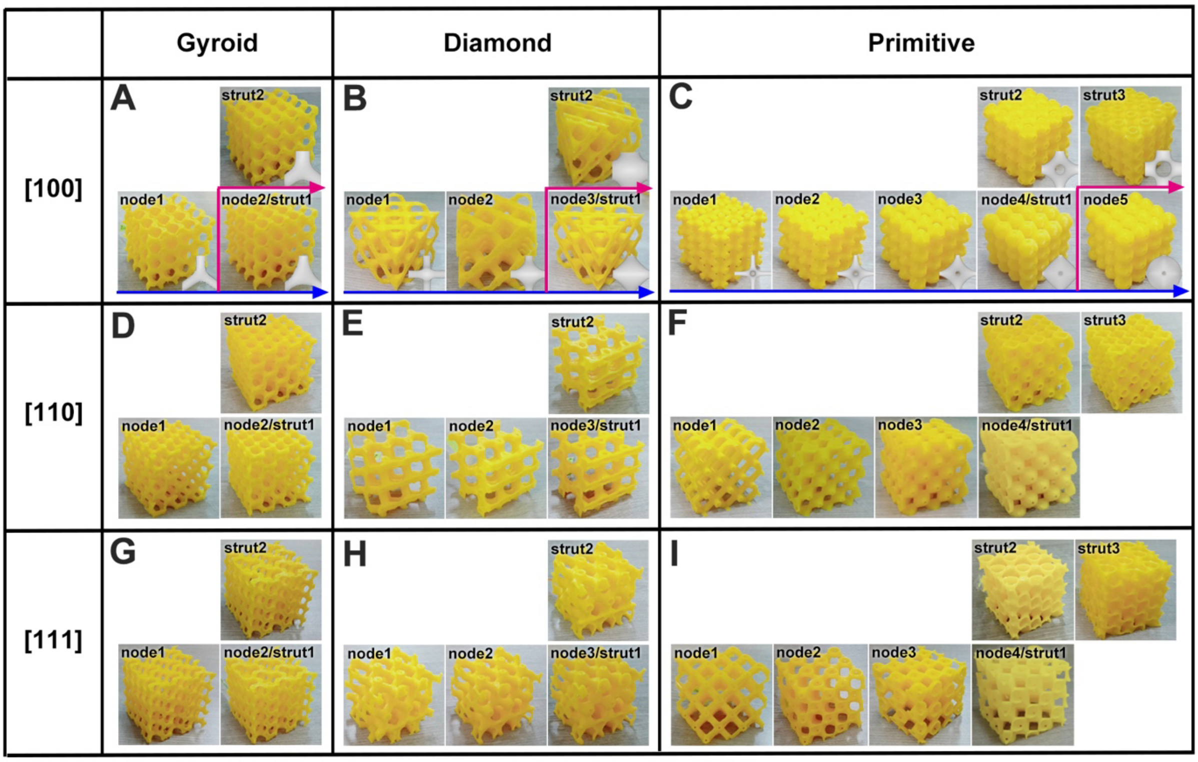

Figure S9. 3D printed models of G-, D- and P-derived structures with a single variable of strut (red arrow path) or node (blue arrow path) size in three characteristic directions. A) P-, B) D- and C) G-derived structures in [100] direction. D) P-, E) D- and F) G-derived structures in [110] direction. G) P-, H) D- and I) G-derived structures in [111] direction. The [110] and [111] directions of P-node5 were not fabricated due to the presence of too many overhanging parts.

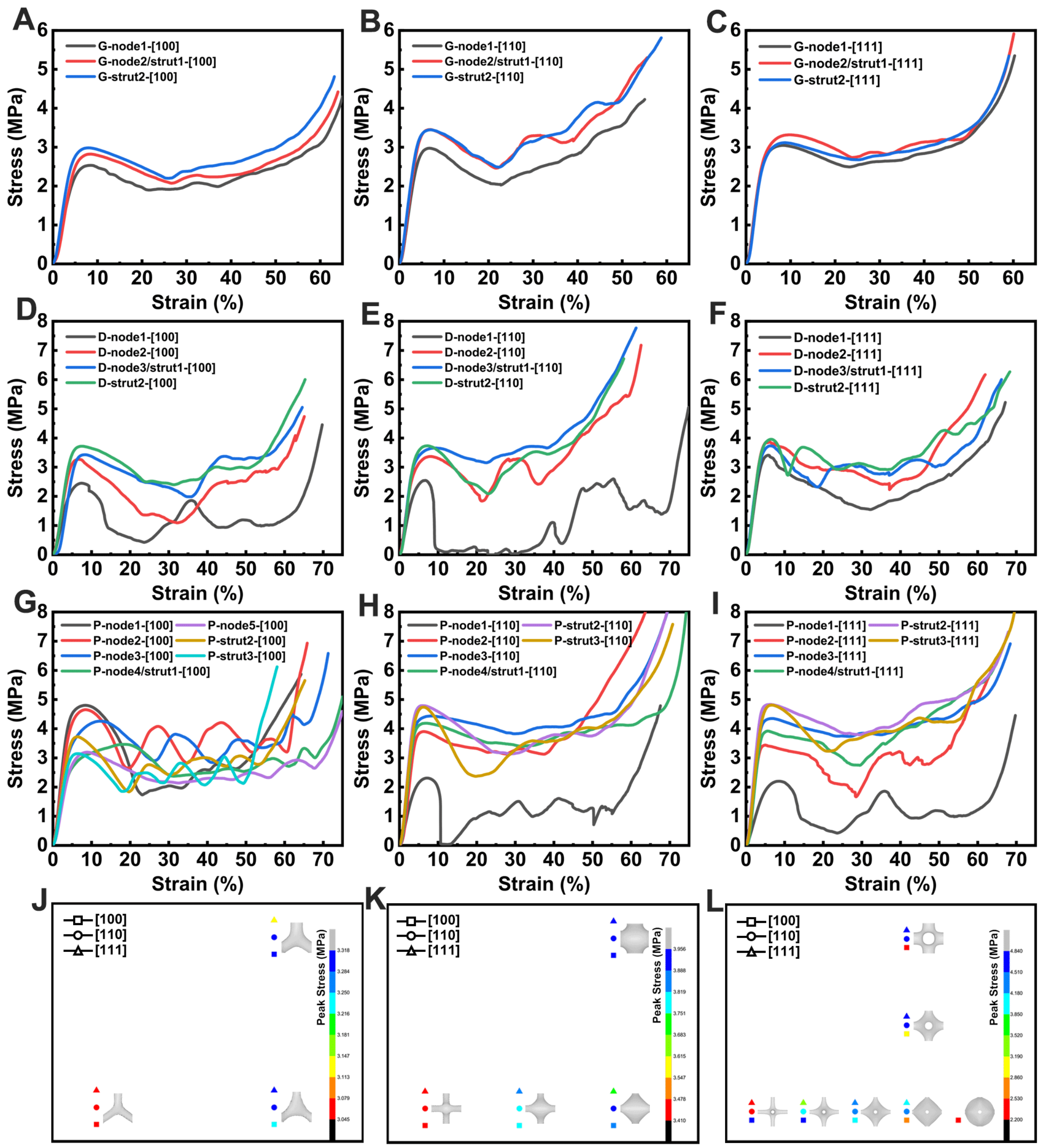

Figure S10. The influence of the node and strut size on the peak stress of G-, D- and P-derived structures in [100], [110] and [111] directions. A) G-derived structures. B) D-derived structures. C) P-derive structures.

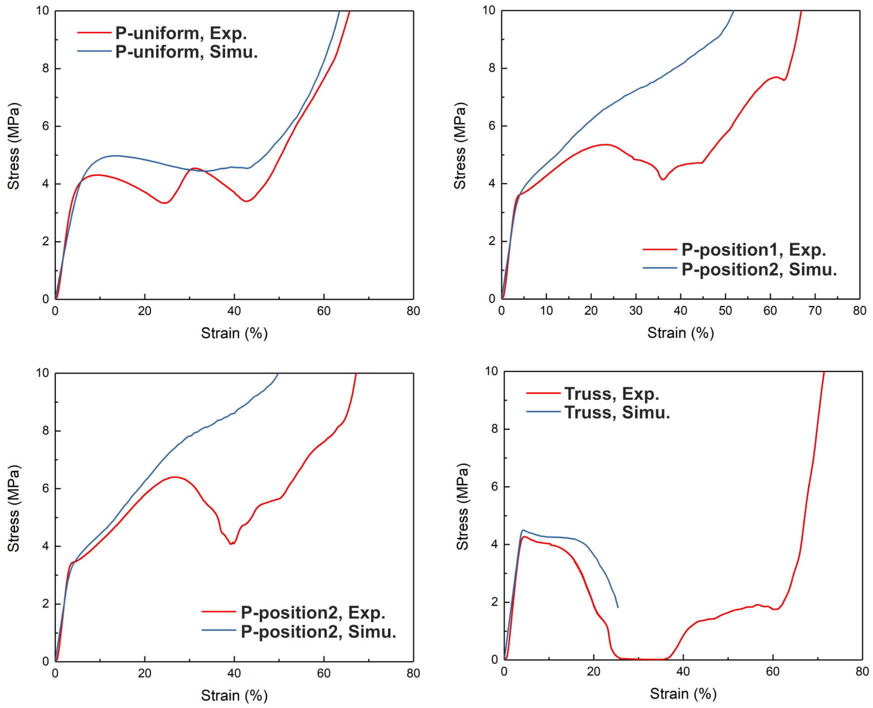

Figure S11. Comparison of stress-strain curves between experimental and FE simulation results for the samples shown in Figure 5.

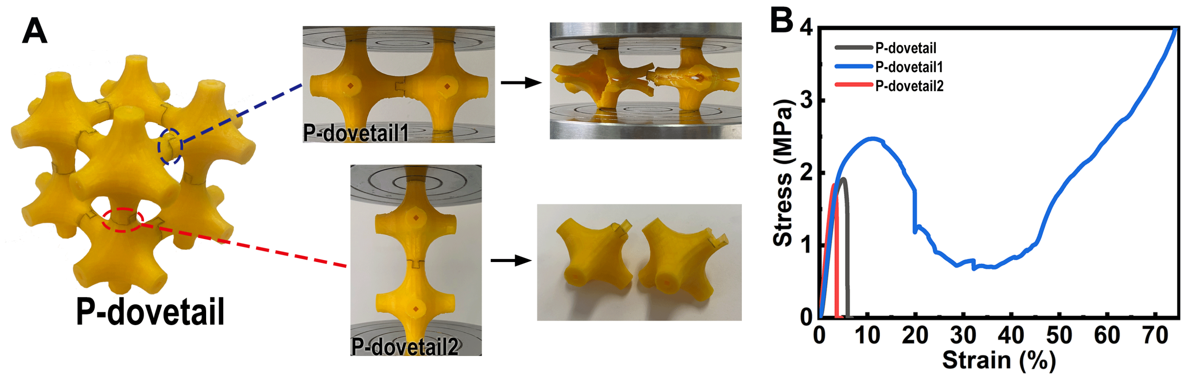

Figure S12. The mechanical properties of dovetail joint measured in vertical and horizontal direction. A) Deformation mechanisms. B) Stress-train curves.

Table S1. The Fourier terms coefficients (c_{hkl}) of novel TPHS structure constructed under pace group symmetry of $Pm\overline{3}m$.

Structures	c_{100}	c_{111}	c_{210}	c_{300}	c_{221}
N-c_{100}-fit	1	/	/	/	/
N-c_{111}-fit	1	-0.00956335	/	/	/
N-c_{111}-0.3	1	-0.3	/	/	/
N-c_{111}-0.3334	1	-0.3334	/	/	/
N-c_{111}-0.478	1	-0.478	/	/	/
N-c_{111}-2	1	-2	/	/	/
N-c_{210}-fit	1	-0.00956335	0.00959441	/	/
N-c_{210}-0.4	1	-0.00956335	0.4		
N-c_{210}-1.0	1	-0.00956335	1	/	/
N-c_{210}-1.4	1	-0.00956335	1.4	/	/
N-c_{210}-4.0	1	-0.00956335	4	/	/
N-c_{300}-fit	1	-0.00956335	0.00959441	-0.0181364	/
N-c_{300}-0.4	1	-0.00956335	0.00959441	-0.4	/
N-c_{300}-8.0	1	-0.00956335	0.00959441	-8.0	/
N-c_{300}-40.0	1	-0.00956335	0.00959441	-40	/
N-c_{221}-fit	1	-0.00956335	0.00959441	-0.0181364	-0.0292538
N-c_{221}-0.3	1	-0.00956335	0.00959441	-0.0181364	-0.3
N-c_{221}-0.9	1	-0.00956335	0.00959441	-0.0181364	-0.45

Table S2. Parameters (Fourier term coefficient c_{hkl} and threshold values t) of G-, D- and P-derived structures.

Structures	C_{100}	C_{110}	C_{111}	C_{222}	C_{331}	C_{511}	t
G-t_0	/	1	/	0.04	/	/	0
G-$t_{0.48}$	/	1	/	0.04	/	/	0.48
G-$t_{1.08}$	/	1	/	0.04	/	/	1.08
D-t_0	/	/	1	/	0.25	-0.25	0
D-$t_{1.49}$	/	/	1	/	0.25	-0.25	1.49
D-$t_{3.95}$	/	/	1	/	0.25	-0.25	3.95
P-t_0	1	/	-0.35	/	/	/	0
P-$t_{2.11}$	1	/	-0.35	/	/	/	2.10
P-$t_{3.24}$	1	/	-0.35	/	/	/	3.23

Table S3. Parameters of G-derived structures with variable nodes/struts size.

Structures	C_{110}	C_{222}	C_{411}	t
G-node1	1	-0.00382452	0.04	1.405
G-node2/strut1	1	/	/	1.348
G-strut2	1	-0.00382452	0.04	1.322

Table S4. Parameters of D-derived structures with variable nodes/struts size.

Structures	C_{111}	C_{331}	C_{511}	t
D-node1	1	0.25	-0.25	5.64
D-node2	1	0.25	/	4.9
D-node3/strut1	1	/	/	4.039
D-strut2	1	0.25	/	3.5

Table S5. Parameters of P-derived structures with variable nodes/struts size.

Structures	C_{100}	C_{111}	t
P-node1	1	-0.35	3.828
P-node2	1	-0.3	3.56
P-node3	1	-0.1667	2.8
P-node4/strut1	1	/	1.8672
P-node5	1	0.2667	0.374
P-strut2	1	-0.11667	1.8672
P-strut3	1	-0.6667	1.8672

Table S6. Parameters of P-related structures with uniform and non-uniform wall thickness.

Structures	surfaces	c_{111}	t
P-uniform	inner	-0.3	3.66
	outer	-0.3	2.06
P-position1	inner	-0.33	3.98
	outer	-.035	2.64
P-position2	inner	-0.327	4
	outer	-0.3	2.58

Table S7. Parameters of level surface equation of infilled rod-like G, D and P with same external frame.

Structures	orientation	c_{hkl}	t
ET-G	[111]	$c_{222} = 0$	1.46
ET-D	[111]	$c_{331} = 0.2$	3.55
ET-P	[100]	$c_{111} = -0.3$	2.64

Table S8. Parameters of assembled P structures with non-uniform wall thickness.

Structures	surfaces	c_{111}	t
P-dovetail	inner	0.327	4
	outer	0.3	2.58
P-straight1	inner	0.283	3.82
	outer	0.4	2.4
P-straight2	inner	0.283	3.82
	outer	0.4	2.4
P-dowel1	inner	0.283	3.82
	outer	0.4	2.4
P-dowel2	inner	0.283	3.82
	outer	0.2667	2.2
P-leading term	inner	0	3.46
	outer	0	1.44

Reference

[1] P. J. F. Gandy, S. Bardhan, A. L. Mackay, J. Klinowski, *Chem. Phys. Lett.* **2001**, *336*, 187-195.
